# Microscaled proteogenomic methods for precision oncology

**DOI:** 10.1038/s41467-020-14381-2

**Published:** 2020-01-27

**Authors:** Shankha Satpathy, Eric J. Jaehnig, Karsten Krug, Beom-Jun Kim, Alexander B. Saltzman, Doug W. Chan, Kimberly R. Holloway, Meenakshi Anurag, Chen Huang, Purba Singh, Ari Gao, Noel Namai, Yongchao Dou, Bo Wen, Suhas V. Vasaikar, David Mutch, Mark A. Watson, Cynthia Ma, Foluso O. Ademuyiwa, Mothaffar F. Rimawi, Rachel Schiff, Jeremy Hoog, Samuel Jacobs, Anna Malovannaya, Terry Hyslop, Karl R. Clauser, D. R. Mani, Charles M. Perou, George Miles, Bing Zhang, Michael A. Gillette, Steven A. Carr, Matthew J. Ellis

**Affiliations:** 1 Broad Institute of Harvard and Massachusetts Institute of Technology, Cambridge, MA 02142 USA; 2 Lester and Sue Smith Breast Center and Dan L Duncan Comprehensive Cancer Center, Baylor College of Medicine, Houston, TX 77030 USA; 3 Verna and Marrs McLean Department of Biochemistry and Molecular Biology, Baylor College of Medicine, Houston, TX 77030 USA; 4 Siteman Comprehensive Cancer Center and Washington University School of Medicine, St. Louis, MO 63110 USA; 5 NSABP Foundation, Pittsburgh, PA 15212 USA; 6 Department of Biostatistics and Bioinformatics, Duke University Medical Center, Durham, NC 27710 USA; 7 Lineberger Comprehensive Cancer Center, University of North Carolina at Chapel Hill, Chapel Hill, NC 27514 USA; 8 Division of Pulmonary and Critical Care Medicine, Massachusetts General Hospital, Boston, MA 02115 USA

**Keywords:** Biological techniques, Cancer

## Abstract

Cancer proteogenomics promises new insights into cancer biology and treatment efficacy by integrating genomics, transcriptomics and protein profiling including modifications by mass spectrometry (MS). A critical limitation is sample input requirements that exceed many sources of clinically important material. Here we report a proteogenomics approach for core biopsies using tissue-sparing specimen processing and microscaled proteomics. As a demonstration, we analyze core needle biopsies from ERBB2 positive breast cancers before and 48–72 h after initiating neoadjuvant trastuzumab-based chemotherapy. We show greater suppression of ERBB2 protein and both ERBB2 and mTOR target phosphosite levels in cases associated with pathological complete response, and identify potential causes of treatment resistance including the absence of ERBB2 amplification, insufficient ERBB2 activity for therapeutic sensitivity despite ERBB2 amplification, and candidate resistance mechanisms including androgen receptor signaling, mucin overexpression and an inactive immune microenvironment. The clinical utility and discovery potential of proteogenomics at biopsy-scale warrants further investigation.

## Abstract (web summary)

Connecting genomics and proteomics allows the development of more efficient and specific treatments for cancer. Here, the authors develop proteogenomic methods to defining cancer signaling in-vivo starting from core needle biopsies and with application to a HER2 breast cancer focused clinical trial.

## Introduction

Cancer proteogenomics integrates data from cancer genomics and transcriptomics with cancer proteomics to provide deeper insights into cancer biology and therapeutic vulnerabilities. Both by improving the functional annotation of genomic aberrations and through insights into pathway activation, this multi-dimensional approach to the characterization of human tumors shows promise for application to precision oncology^1–7^

Here we address tissue requirements for proteogenomics, which restricts translational research opportunities and applicability to cancer diagnostics. For example, the Clinical Proteomic Tumor Analysis Consortium (CPTAC) requires at least 100 mg of tumor tissue, which typically yields quantitative information on >10,000 proteins and >30,000 phosphosites per sample^8^. For clinical diagnostics a single snap-frozen tumor-rich core needle biopsy (∼ < 20 mg) must provide sufficient DNA, RNA and protein for deep-scale proteogenomic profiling. To reduce these tissue requirements, we now describe methods to generate high-quality DNA, RNA and protein for deep-scale DNA and RNA sequencing and proteome and phosphoproteome analysis from a single 14 G core needle biopsy (Biopsy Trifecta Extraction, (BioTExt)) and a microscaled liquid chromatography-mass spectrometry (LC-MS/MS)-based proteome and phosphoproteome analysis pipeline (MiProt) that requires only 25 μg peptide per sample. As technical proof-of-principal, we apply these methods to a pilot study designed to test the feasibility of proteogenomic profiling before and 48–72 h after initiating chemotherapy. We choose trastuzumab-based treatment for ERBB2 + breast cancer as an example of an oncogenic kinase-driven tumor where proteogenomic analyses and pharmacodynamic studies should provide significant insights into variability in treatment outcomes^9,10^.

## Results

### Biopsy Trifecta Extraction protocol

**Fig. 1:**
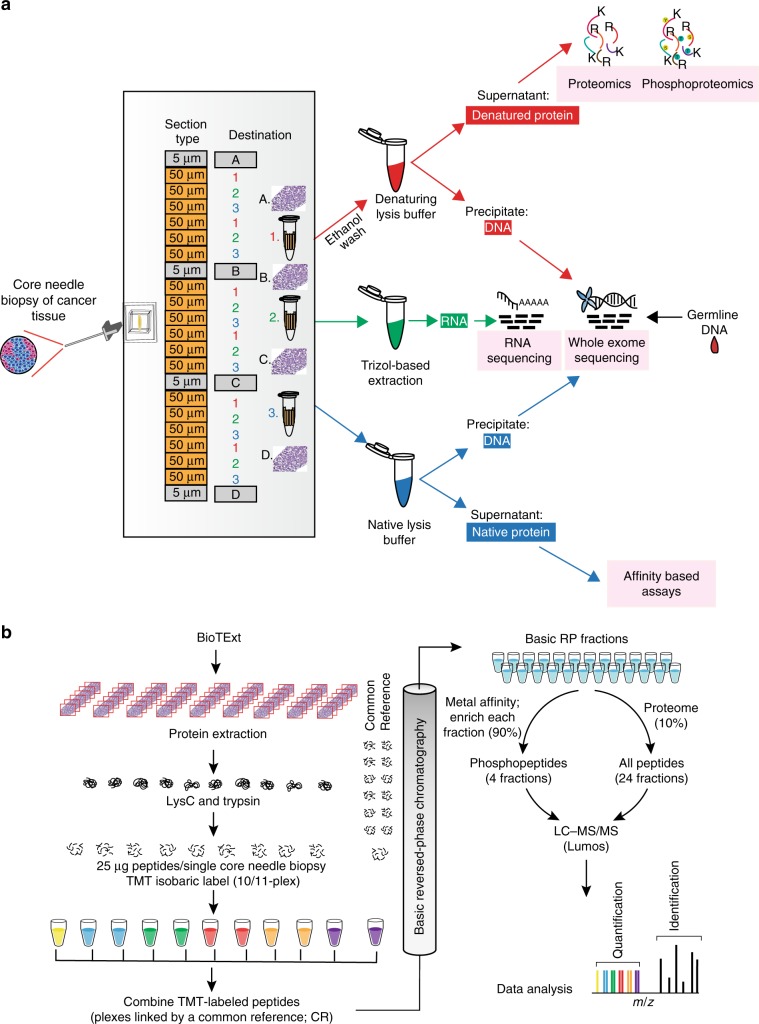
The Biopsy Trifecta EXTraction based proteogenomics workflow. **a** In the Biopsy Trifecta EXTraction (BioTEXT) protocol, patient derived OCT-embedded core needlebiopsies are sectioned, followed up by extraction of DNA, RNA and proteins for deep-scale proteogenomics characterization and by immunohistochemistry-based imaging. **b** The Microscaled Proteomics (MiProt) workflow allows deep-scale proteomics and phosphoproteomics characterization with 25 μg of peptides per core-needle biopsy. MiProt uses a common reference that could be used for comparison across all samples within a single-TMT10/11 plex and across several TMT10/11 plexes spanning several core biopsies.

To microscale specimen processing an optimal cutting temperature (OCT)-embedded core biopsy is serially sectioned with alternating 50um sections transferred into 3 different 1.5 ml tubes (Fig. 1a). A total of six sections were transferred into each tube. To assess sample quality, 5 μm sections were taken before the first and after every sixth 50 μm section for H&E staining. 50% average tumor content was required for further analysis. The first tube was used to extract denatured protein and DNA, the second for RNA isolation, and the third for native protein and DNA. The denatured protein was subsequently used for proteomic and phosphoproteomic analyses described herein, and the DNA and RNA was used for genomic analyses. The native protein analyses will be described elsewhere.

### Development and evaluation of a microscaled proteomics protocol

To initially assess analytes generated by BioTExt, OCT-embedded core-needle biopsies were collected from two basal-like breast cancer patient-derived xenograft (PDX) models (WHIM2, WHIM14) and two luminal models (WHIM18 and WHIM20)^11^. The biopsy yields ranged from 2.5–14 μg DNA, 0.9–2.3 μg RNA and 280–430 μg of protein. Extraction yields for nucleic acids are provided in Supplementary Fig. 1A. Because needle sizes vary (14–22 gauge), a low minimum of 25 μg of input peptide/sample was set as the target for proteomics using a tandem mass-tagging (TMT) peptide labeling approach^12^ (Fig. 1b). Since the mass tags are isobaric, signals from the same peptides in each sample stack at the MS1 level, improving overall sensitivity for identification and quantification, a key advantage at this input scale. Multiplexing also increases sample analysis throughput by 10-fold relative to label-free approaches. Successful microscaling required several modifications to the bulk-optimized CPTAC workflow^8^ to allow low input profiling. This overall method is referred to as Microscaled Proteomics (MiProt).

**Fig. 2:**
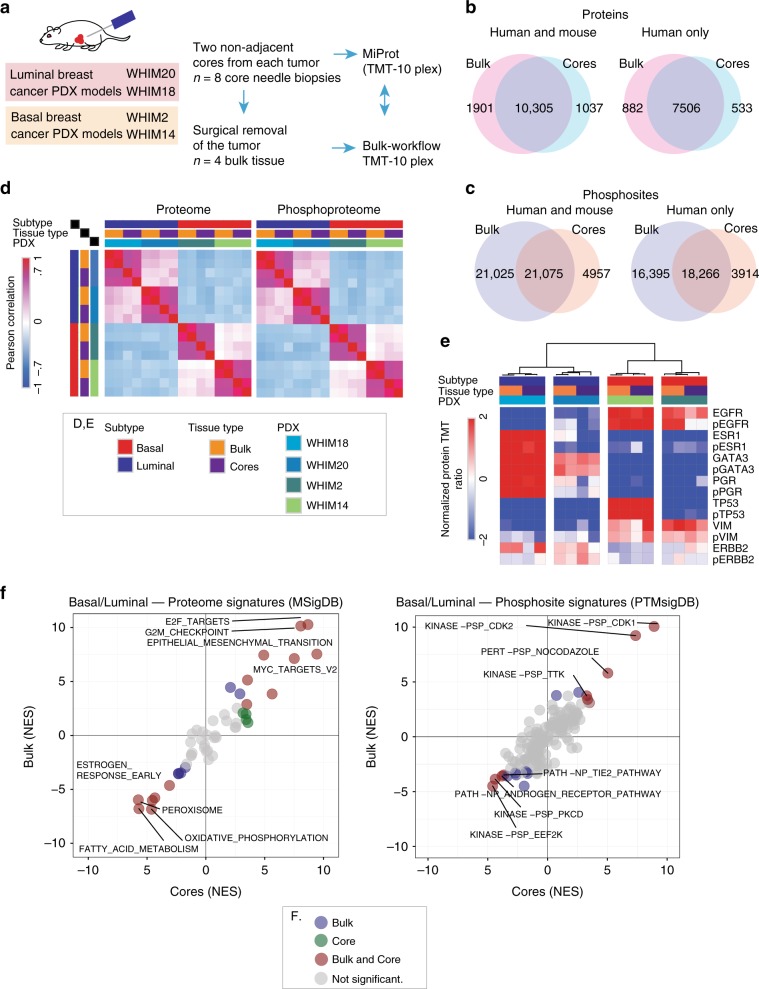
Evaluation of the BioText and MiProt workflow on preclinical PDX models. **a** Non-adjacent, core needle biopsies were collected from 2 basal and 2 luminal PDX models followed by surgical removal of tumors. Proteomic and phosphoproteomic characterization of cores was performed using the MiProt workflow, and the bulk tissue was characterized using the CPTAC workflow described in Mertins et al^8^. **b** Venn-diagram shows the number of overlap between human and mouse or human proteins quantified in cores and bulk tissue. **c** Venn-diagram shows the overlap between human and mouse or human phosphosites. **d** Pearson correlation of TMT ratios for proteins (left) and phosphosites (right) between each sample from both cores and bulk across all 4 PDX models. **e** The heatmap shows the TMT ratios for key differentially regulated Luminal vs. Basal breast cancer associated proteins and phosphoproteins (average expression of identified phosphosites) identified across both bulk and cores experiments. **f** Gene-centric and phosphosite-centric pathway or kinase activity enrichment analysis was performed using GSEA (MSigDB “Cancer Hallmarks”, left) and PTM-SEA (PTMSigDB, right), respectively, for Luminal-Basal differences captured in bulk (*y*-axis) and core (*x*-axis) tissue. limma derived signed Log10 *p*-values were used to pre-rank differential features for both GSEA and PTM-SEA analysis. The pathway/phospho-signatures that are significant in both cores and bulk are indicated in brown.

The PDX material was used to determine if the proteomic coverage for core-needle biopsies is comparable to those obtained using a workflow optimized for bulk tumors (the Clinical Proteomics Tumor Analysis Consortium (CPTAC) workflow)^8,11^ (Fig. 2a). Two needle-biopsy cores were collected from each PDX model. The cored xenograft tumors were then surgically removed for bulk material analysis. The cores were OCT-embedded, flash frozen and subjected to BioTExt followed by MiProt. The remaining bulk tumors were flash frozen, cryopulverized and analyzed using the original CPTAC workflow^8,13^. Totals of 300 μg of peptides per sample were analyzed with the original CPTAC workflow and 25 μg of peptides per sample with the MiProt workflow using a randomized experimental layout (Supplementary Data 1). Protein and phosphosite expression is reported as the log ratio of each sample’s TMT intensity to the intensity of an internal common reference included in each plex. Both workflows identified more than 10,000 proteins, of which >7500 were identified as human. Extensive overlap was observed between the populations of proteins identified by the two approaches (Fig. 2b, Supplementary Data 2A). MiProt identified >25,000 phosphosites from each core, and these sites show substantial overlap with those identified by bulk CPTAC workflows (Fig. 2c, Supplementary Data 2B, Supplementary Fig. 2A). The identification of over 25,000 phosphosites by the MiProt method is of particular note as this is less than a two-fold reduction in quantified sites relative to the CPTAC bulk workflow^8^ despite using 12-fold less tumor material per sample. While prior studies have reported relatively high numbers of proteins (~4500) from small amounts of tissue material^14^, the very large number of phosphosites we obtain using just 25 μg of peptide/sample has not been described previously. There was a high correlation of TMT ratios between replicates of bulk tumors and between replicates of cores across all 4 PDX models for both the proteomics and phosphoproteomics data (Fig. 2d, Supplementary Fig. 2B). In addition to a high degree of overlap in protein and phosphosite identities, expression was also highly correlated (Pearson correlation, *R* > 0.65) between cores and bulk for individual PDX models, as can be visualized by the close juxtaposition of core and bulk samples from the same PDX model upon unsupervised hierarchical clustering (Supplementary Fig. 2C).

Expression profiles of key basal and luminal markers show a comparable trend overall between bulk and core samples (Fig. 2e) at the levels of both the proteome and phosphoproteome for all PDX models except for WHIM20, where phosphorylated EGFR, phosphorylated PGR and ESR1 protein show reduced expression in cores relative to bulk, suggesting that there may be some heterogeneity in this particular PDX model^11^. By contrast, ERBB2, a breast cancer marker that should not be highly expressed in these clinically ERBB2- (no *ERBB2* amplification) cases showed more uniform expression across the different PDX models. Overall, cores provided proteomics data that yielded results consistent with those obtained from global expression profiles from bulk tissue.

To address whether differentially regulated pathways and phosphosite-driven signaling in luminal vs. basal subtypes were captured by the microscaled workflow, pathway-level and kinase-centric analyses were applied to the bulk and core sample data. Single-sample gene-set enrichment analysis (ssGSEA) was applied to proteomics data, and post-translational modifications set enrichment analysis (PTM-SEA) to the phosphoproteomic data^15,16^. The luminal-basal differences captured by bulk tissue analysis were highly correlated with differences detected using cores for both protein and phosphosite expression (Fig. 2f, Supplementary Data 2C, D). Of note, the data recapitulates previously observed luminal-basal differences and provided a quality metric for the proteomics dataset both for cores and bulk tissue^2,6^. The same conclusion was reached in bulk vs. core comparisons performed on the normalized TMT protein ratios for individual PDX models (Supplementary Fig. 2D). Despite identifying ~40% fewer phosphorylation sites, most of the differential Luminal-Basal kinase signatures identified in the bulk tissue were captured by MiProt (Fig. 2f, right).

### Microscaled proteogenomic analyses applied to clinical cores

The PDX-based preliminary data encouraged the application of these methods to a pilot proteogenomics breast cancer study (Discovery protocol 1 (DP1); NCT01850628). The aim of DP1 was to investigate the feasibility of proteogenomic profiling in core biopsies from patients with locally advanced ERBB2 + breast cancer. Patients were treated at the physicians’ discretion, typically with trastuzumab in combination with pertuzumab and chemotherapy. The protocol was designed to study acute treatment perturbations by accruing samples before and 48 to 72 h after treatment (referred to pre-treatment and on-treatment, respectively, throughout the text).

**Fig. 3:**
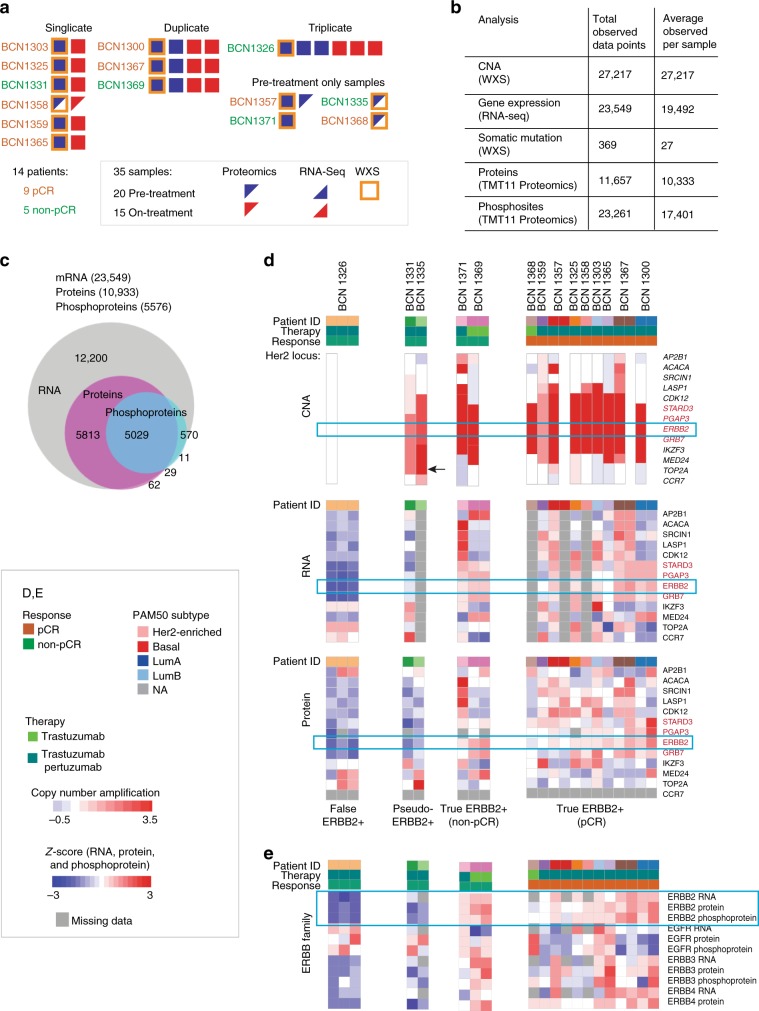
Microscaled proteogenomics of the DP1 clinical trial. **a** Overview of proteogenomics samples obtained from pre- and on-treatment core biopsies from the DP1 clinical trial. Each block indicates the data obtained from a separate core. **b** Microscaled proteogenomics achieves a high level of proteogenomics depth for the DP1 core needle biopsies. Table summarizing total proteogenomics coverage and numbers of mutated genes for all samples and average coverage across all analyzed cores is shown on the right. **c** The Venn-diagram shows the overlap between all genes identified across RNA-seq, proteomics and phosphoproteomics. **d** Heatmap summarizing proteogenomics features of the *ERBB2* amplicon and adjacent genes at the level of CNA, RNA and protein expression. The set of genes in red make up the core of the *ERBB2* amplicon and showed consistently high copy number amplification, RNA, and protein levels in all of the pCR cases (True ERBB2+ pCR set on the right) and in BCN1371 and 1369 (True ERBB2 + non-pCR set) but significantly lower protein levels in BCN1326 (False ERBB2+) and BCN1331 and BCN1335 (Psuedo ERBB2 + set). The arrow points to the amplified *TOP2A* gene in BCN1335. **e** The heatmap at the bottom shows corresponding *Z*-scores of RNA, protein, and phosphoprotein expression of ERBB1-4 across all 14 patients. ERBB3 protein and phosphoprotein and ERBB4 protein levels were also significantly lower in BCN1326, BCN1331 and BCN1335 than in the set of pCR cases.

As shown in the REMARK (Reporting Recommendations for Tumor Marker Studies)^17^ diagram (Supplementary Fig. 3), core biopsy samples were available from 19 patients. Proteogenomic analysis could be conducted on samples from 14 patients as five cases showed tumor content <50%. Analyte yield varied across different cores, but the lower-range yields of DNA, RNA and protein (0.4 μg, 0.2 μg and 45 μg, respectively) were sufficient to demonstrate the suitability of the optimized extraction protocol for clinical biopsy specimens (Supplementary Fig. 1B). Protein, and RNA when available, were also analyzed for on-treatment cores from 10 patients, with analysis of duplicate pre- and on-treatment cores achieved in four of the patients, and of triplicate cores in one patient (Fig. 3a). In total, 35 cores were analyzed. Tumor and germline whole-exome sequencing was performed using DNA from a single baseline core for all 14 patients. DNA isolated from cores using BioTExt yielded target coverage comparable to that from genomic DNA isolated from blood (generated using standard organic extraction techniques) (Supplementary Fig. 4A). RNA sequencing was successful for 30 cores corresponding to 11 of the 14 patients, and MiProt analysis was successful in all 35 available cores.

On average, we obtained copy number information on >27,000 genes, measured mRNA transcripts for >19,000 genes, and quantified >10,000 proteins and >17,000 phosphosites from each sample, with a large overlap of gene identification across different datasets (Fig. 3b, c, Supplementary Data 3, 4). The coverage depth was similar to that obtained in previous large-scale breast cancer proteogenomics efforts with the exception of the phosphoproteome coverage, which achieved about half of the number of sites previously reported for tumor bulk-level characterization^2,18^. For 13 out of the 14 cases, *ERBB2* amplification was confirmed by exome sequencing along with amplifications and mutations in a range of genes previously implicated by the TCGA and ICGC breast cancer studies (Supplementary Fig. 4B)^19,20^. We also observed a similar overall pattern of structural variation (Supplementary Fig. 4C), including amplifications of chromosomes 1, 8 and 20^20^. The median gene-wise Spearman correlation between mRNA and protein across patient cores (*n* = 11) was 0.38, consistent with previous bulk-focused proteogenomics studies (Supplementary Fig. 4D)^1,2,4,18^. In addition, co-expression networks derived from MiProt protein expression better predicted KEGG pathway function than those derived from mRNA expression for a similar proportion of pathways as previously reported for the published CPTAC breast cancer cohort^21^ (Supplementary Fig. 5A). An assessment of BioTExt sample processing reproducibility was afforded by duplicate and pre- and on-treatment cores from the same patient. Unsupervised hierarchical clustering based on 500 most-variable genes resulted in all duplicate cores clustering together at the level of mRNA, protein and phosphosite expression with the exception of samples from case BCN1365, where pre- and on-treatment profiles did not cluster together at the level of phosphosite expression (Supplementary Fig. 5B).

### Proteogenomic analysis of the *ERBB2* locus

A pathological Complete Response (pCR) occurred in 9/14 cases (64%), and 5 patients had residual cancer at surgery (non-pCR). To probe the possibility that some of the non-pCR cases were due to misassignment of ERBB2 status, proteogenomic analysis of the region of chromosome 17q spanning the *ERBB2* locus and adjacent genes was performed (Fig. 3d). Most obviously, exome sequencing of BCN1326 did not show amplification of *ERBB2* or other nearby genes (Fig. 3d, upper panel) and exhibited markedly lower levels of ERBB2 RNA (Fig. 3d, middle panel) and protein expression (Fig. 3d, lower panel) than pCR cases, suggesting a false positive (False ERBB2+). Expression levels from genes immediately flanking *ERBB2* (*STARD3, PGAP3 and GRB7*, highlighted in red in Fig. 3d) were also lower than in pCR cases functionally confirming a lack of amplification-driven gene dysregulation. BCN1331 and BCN1335 may represent a more subtle form of false positivity. While these samples showed a gain of *ERBB2* copy number, ERBB2 protein levels remained low, similar to BCN1326. BCN1335 showed greater absolute amplification of *TOP2A* than of *ERBB2* (see black arrow Fig. 3d upper panel), and the TOP2A protein was markedly over-expressed compared to all other cases (Fig. 3d lower panel, of note the RNA analysis failed in this sample). This suggests that TOP2A was a potential alternative driver. Levels of STARD3, PGAP3 and GRB7 both for RNA (BCN1331) and protein (BCN1331 and BCN1335) were also low in BCN1331 and BCN1335. Cases with ERBB2 gene copy gain without focal overexpression are referred to as pseudo ERBB2 positive (pseudo ERBB2+). When comparing BCN1326, BCN1331 and BCN1335 as a group with the nine true ERBB2 + pCR cases, both the arithmetic mean for STARD3, ERBB2 and GRB7 protein log TMT ratios and the protein log ratios of each gene separately were significantly lower (two-sample T-test; arithmetic mean: *p* = 0.0114, STARD3: *p* = 0.0255, ERBB2: *p* = 0.0073, GRB7: *p* = 0.0399; *n* = 3 false and pseudo ERBB2 + non-pCR cases, *n* = 9 true ERBB2 + pCR cases). Protein levels of ERBB2 dimerization partners ERBB3 and ERBB4, as well as phospho-ERBB3, were also significantly under-expressed (two-sample T-test; ERBB3 protein: *p* = 0.0097, ERBB3 phosphoprotein: *p* = 0.0318, ERBB4 protein: *p* = 0.0131; *n* = 3 false ERBB2 + non-pCR cases, *n* = 9 true ERBB2 + pCR cases) (Fig. 3e). In contrast, protein and phosphoprotein levels of EGFR, the remaining dimerization partner of ERBB2, does not appear to be correlated with ERBB2 levels and was high in the non-pCR samples, suggesting that EGFR homodimers could play a driver role in signaling when ERBB2 is low^22,23^. A central immunohistochemistry analysis indicated that ERBB2 was 1+ in BCN1326 and 2+ in BCN1335 and BCN1331, while all the pCR cases were assigned 3+ staining (Supplementary Fig. 6A). Supporting the quantitative potential of microscaled proteomics, the samples with an IHC score of 3+ in the central analysis showed significantly higher levels of ERBB2 expression than tumors scored 1+ or 2+ (Wilcoxon two-sample rank sum test with *n* = 5 for IHC 1+ or 2+ and *n* = 15 for IHC 3+, *p* = 0.00013). Parallel reaction monitoring (PRM) was also deployed as an orthogonal label-free protein quantification method on the same samples with an excellent correlation (Spearman Rho = 0.92, *p* *=* 0, *n* = 32) between the TMT and PRM-based MS approaches (Supplementary Fig. 6B-D).

### Phosphoproteomic analysis of acute on-treatment samples

**Fig. 4:**
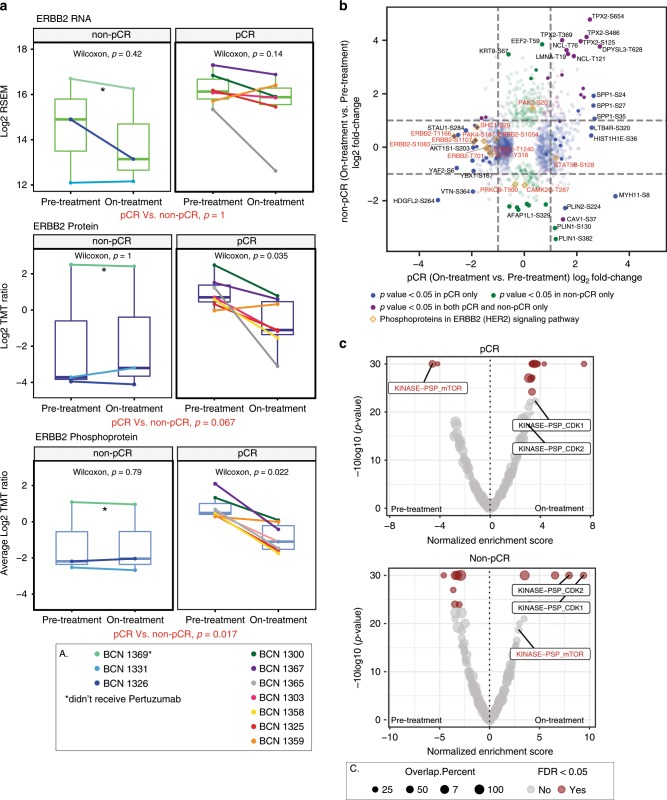
Downregulation of ERBB2 and mTOR signaling in cases with pCR. **a** Effect of anti-ERBB2 treatment on ERBB2 RNA, protein, and phosphoprotein levels for each patient with on-treatment data. *p*-values were calculated by paired Wilcoxon signed rank tests for on-treatment vs. pre-treatment ERBB2 expression for each group. The pCR vs. non-pCR *p*-values are derived from Wilcoxon rank sum tests comparing log2 fold changes of on-treatment to pre-treatment levels from pCR patients to those from non-pCR patients. For patients with multiple cores, the mean expression value was used. *n* = 3 for all non-pCR; *n* = 6 for pCR RNA or *n* = 7 for pCR protein and phosphoprotein. Boxplots are centered on the median and show first and third quartiles for each group. Asterisk indicates patient BCN1369 that didn’t receive Pertuzumab. **b** Scatter plot showing differential regulation of individual phosphosites before and after treatment in pCR and in non-pCR cases. Shown are the on-treatment vs. pre-treatment log2 fold changes in non-pCR (*y*-axis) vs. the log2 changes in pCR samples (*x*-axis) for phosphosites with *p*-value < 0.05 by limma analysis of differential expression in either group (*n* = 7 for pCR; *n* = 2 for non-pCR). Blue and green circles indicate phosphosites in pCR and non-pCR, respectively that show significant differential regulation in either group alone. Purple circles indicate significantly regulated phosphosites in both sets of patients. The orange diamond outlines highlight phosphosites on proteins in the KEGG ErbB signaling pathway (hsa04012). The transparency of each point reflects its significance after BH-adjustment (adjusted *p* < 0.05 is solid, and more transparent points have higher adjusted *p*-values). **c** PTM-SEA was applied to the signed -Log10 *p*-values from limma differential expression analysis of on- vs. pre-treatment phosphosite levels from pCR (upper panel) and non-pCR (lower panel) cases. The volcano plots show the Normalized Enrichment Scores (NES) for kinase signatures. Brown circles indicate signatures with significant FDR ( < 0.05).

To test the feasibility of proteogenomics for the study of treatment perturbations, pre- and on-treatment core biopsies for nine patients with pCR and three patients without pCR were studied. Differential treatment-induced changes were not observed at the RNA level (Fig. 4a), and, while the ERBB2 protein levels showed significant reduction in pCR cases (paired Wilcoxon signed rank test, *p* = 0.031, *n* = 7 for pCR cases), the two-sample rank Wilcoxon rank sum *p*-value for the comparison of this reduction between pCR (*n* = 7) and non-pCR (*n* = 3) cases was not significant (*p* = 0.067). However, greater downregulation of ERBB2 phosphoprotein (mean of all ERBB2 phosphosites) levels after 48–72 h in pCR cases than in non-pCR cases was observed (two sample Wilcoxon rank sum test, *p* = 0.017, *n* = 3 for non-pCR cases, *n* = 7 for pCR cases; Fig. 4a). To explore these data further, limma^24^, a more advanced statistical method specifically designed for differential expression analysis of small sample size studies, was employed. (Supplementary Data 5). Differential ERBB2 RNA expression was again not seen for any comparison (Supplementary Fig. 7). However, there was significant pCR-specific downregulation for both ERBB2 protein (limma; *p* = 0.002, *n* = 7 for pCR; *p* = 0.63; *n* = 2 for non-pCR; and *p* = 0.029 for pCR, *n* = 7, vs. non-pCR, *n* = 2) and phosphoprotein levels (*p* = 0.000014, *n* = 7 for pCR; *p* = 0.88, *n* = 2 for non-pCR; and *p* = 0.0086 for pCR, *n* = 7, vs. non-pCR, *n* = 2) (Supplementary Data 5). Differential analysis of individual phosphorylation sites also reveal pCR-specific significant downregulation of several phosphosites on proteins from the ERBB2 pathway, including sites on ERBB2 and SHC1, an adaptor that binds to ERBB2^25^ (Fig. 4b). Most of the significant changes that were at least 2-fold and those that affected the ERBB2 pathway were observed in the site-level phosphoproteomics data (Fig. 4b; Supplementary Fig. 7).

Given the well-understood kinase signaling cascades downstream of ERBB2^22^, a recently published tool for pathway analysis of phosphosites, PTM-SEA, was applied to the phosphoproteomics data^15^ (PTMsigDB: https://github.com/broadinstitute/ssGSEA2.0). Figure 4c shows significant phosphosite signatures for the comparisons tested (on- vs pre-treatment changes in pCR only and in non-pCR only) (Supplementary Data 6). Supplementary Fig. 8 shows a heatmap of phosphoproteome driven signatures that were significantly differentially regulated (FDR < 0.05) upon treatment in either of the two groups. While the inferred activities of CDK1 and CDK2 kinases (KINASE−PSP_CDK1, KINASE−PSP_CDK2) were upregulated in the non-pCR patients, downregulation of mTOR activity (KINASE-PSP_mTOR) was most prominently exclusive to the pCR cases upon treatment.

### Exploration of response features in individual non-pCR cases

**Fig. 5:**
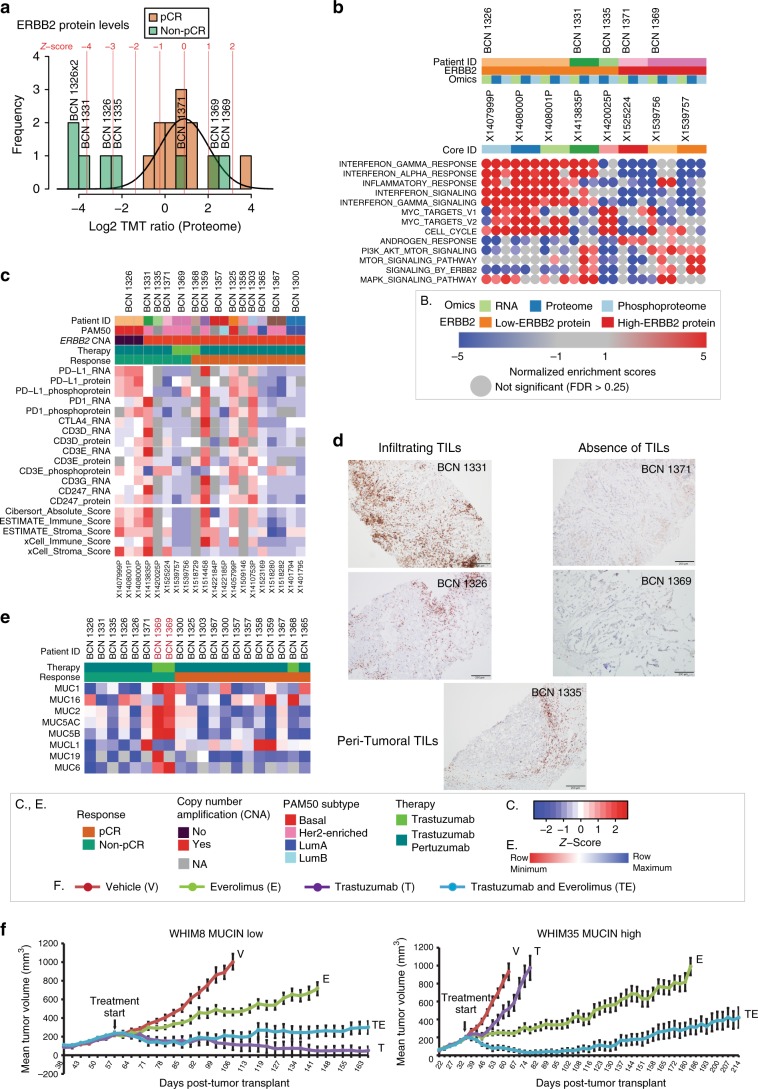
Proteogenomics analysis of baseline untreated non-pCR cases. **a** Outlier analysis was performed to identify differentially regulated mRNA, proteins or phosphoproteins in each pre-treatment sample from non-pCR cases relative to the set of pre-treatment samples from all pre-treated pCR cases. Shown is the ERBB2 protein distribution across all patients; brown and green bars indicate the frequencies for each protein level bin in pCR and non-pCR cores, respectively. The line shows the normal distribution of pCR samples from which the *Z*-score for each non-pCR sample was derived. Corresponding *Z*-scores levels are indicated in red. **b** Heatmap showing normalized enrichment scores (NES) from single sample Gene Set Enrichment Analysis (ssGSEA) of outlier *Z*-scores from non-pCR cases. Shown are a subset of differentially regulated pathways with false-discovery rate less than 25% (FDR < 0.25). **c** Heatmap showing expression levels of key immune-checkpoint and T-cell marker (CD3) genes and of RNA based immune and stroma scores from ESTIMATE, Cibersort, and xCell. **d** Photomicrographs showing anti-CD3 immunohistochemical staining profiles of non-pCR cases (original magnification: 20 × ). **e** Heatmap showing Mucin protein expression across all pre-treated patients. **f** WHIM8 and WHIM35 PDX models were treated with vehicle, trastuzumab, everolimus or the combination of trastuzumab and everolimus. The graph shows the mean-tumor volume at several timepoints (*N* = 15 (before 1 week), *N* = 12 (1 week to 4 weeks), *N* = 9 (after 4 weeks)) after tumor implantation and subsequent treatment, and error bars show standard error of mean.

To explore candidate biological processes that may contribute to inadequate response to therapy in non-pCR cases, RNA, protein and phosphoprotein outlier analyses on data from each pre-treatment core from the non-pCR cases with respect to the set of pre-treatment pCR cores were performed. Specifically, *Z*-scores were calculated for each gene/protein in a given individual non-pCR core relative to the distribution established from all of the pre-treatment pCR cores. The *Z*-scores of ERBB2 protein expression in non-pCR cases were consistent with the observations noted above; ERBB2 RNA, protein and phosphoprotein levels in patients BCN1326, BCN1331 and BCN1335 were outliers with negative *Z*-scores while ERBB2 expression in patients BCN1369 and BCN1371 lay within the normal distribution of the pCR cases (Fig. 5a, Supplementary Fig. 9). *Z*-scores derived from the outlier analysis for each of the data points (RNA, proteome and phosphoproteome; see Supplementary Data 7A) were used for single sample Gene Set Enrichment Analysis (ssGSEA). Figure 5b highlights a subset of immune-centric and oncogenic signaling pathways that show differential enrichment in the non-pCR cases. The expanded list is available as Supplementary Data 7B. Consistent, significantly enriched pathway-level differences across replicate cores and multiple data types from a single patient was observed (Fig. 5b), confirming that microscaled proteogenomics data obtained from cores in a clinical setting yield reproducible results. Interestingly, distinct biological pathways show differential enrichment in each of the individual non-pCR cases relative to the pCR class (Fig. 5b, Supplementary Data 7B).

Of the complex patterns revealed by differential pathway analysis, immune-related and interferon signaling pathways showed consistent upregulation across the data sets in samples from two of the three cases with lower expression of ERBB2, BCN1326 and BCN1331. In contrast, these pathways show variable downregulation for the remaining non-pCR cases. To further explore these findings, the expression of T-cell receptor (CD3 isoforms and CD247) and immune checkpoint (PD-L1, PD1, and CTLA4) genes were analyzed and immune profiles from the RNA-seq data were generated using established tools (Cibersort, ESTIMATE, and xCell). Examination of immune profile scores and of expression of T-cell receptors and targetable immune checkpoint regulators supported the presence of an active immune response in BCN1326 relative to other samples (Fig. 5c, Supplementary Fig. 10). Similarly, immune profile scores also indicate that BCN1331 had an activated immune microenvironment, and PD1 RNA expression was higher in this patient than in any other case (Fig. 5c). The five non-pCR cases were stained for the pan T-cell marker CD3 to validate these proteogenomic findings (Fig. 5d). Consistent with the active immune microenvironment (Fig. 5c), both BCN1326 and BCN1331 demonstrate tumor T-cell infiltration. In contrast, a predominant peri-tumoral or “immune-excluded” inflammatory reaction was observed in BCN1335, and a complete paucity of T-cells (immune-desert) was observed in the two resistant proteogenomically confirmed ERBB2 + cases, BCN1371 and BCN1369, consistent with the lack of immune signaling (Fig. 5b).

Other variable differential features in resistant cases included PI3K/AKT/mTOR and MAPK signaling, all of which represent potential therapeutic opportunities. ERBB2 pathway activation in BCN1331 is unexpected given the very low level of ERBB2 protein but could be explained by expression of EGFR or phosphorylated EGFR (Fig. 3e). MYC targets were consistently upregulated at the protein and phosphoprotein levels in BCN1326 and BCN1335, and the androgen response pathway was upregulated at all levels in BCN1371. Consistent with the elevated AR signaling observed in BCN1371 (Fig. 5b), this tumor exhibits histologic features of an apocrine cancer with intensely eosinophilic cytoplasm and AR expression (Supplementary Fig. 11 middle and lower panel). Interestingly, BCN1331 also expressed AR by IHC without activation of an androgen response signature or apocrine features (Fig. 5b, Supplementary Fig. 11), consistent with the disconnect between AR expression and AR signaling in breast cancer noted by others^26^. Also, for patient BCN1371, we did not observe significant upregulation of PI3K signaling (Fig. 5b) despite *PIK3CA* mutation (E545K), consistent with the disconnect between *PIK3CA* mutation and effects when signaling assessed by reverse phase protein array (RPPA)^19,27^. Table 1 summarizes the proteogenomic features observed for each non-pCR case.

**Table 1:** Summary of proteogenomic features from non-pCR cases.

Patient ID	RNAseq-PAM50 subtype and ER status	*ERBB2* amplicon structure by WES	Druggable mutations	Differential pathways (relative to pCR cases) UP (upregulated), DOWN (downregulated)	TILs and immune microenviroment proteomics	Treatments received
BCN1326	Basal ER+	*ERBB2* not amplified Low ERBB2 expression		MYC UP Mitosis UP Interferon signaling UP	Infiltrating TILs PDL1 high Phospho-PD1 high	Doc, CP, T, P
BCN1331	HER2-E ER+	Broad lower-level *ERBB2* amplification Low ERBB2 expression (pseudo-amplified)		ERBB2/3 UP MAPK UP PI3K UP mTOR UP Interferon signaling UP	Infiltrating TILs PD1 RNA and Phospho-PD1 high	Doc, CP, T, P
BCN1335	RNA failure ER+	Amplicon driving *TOP2A* Low ERBB2 expression (pseudo-amplified)	*BRCA1*, R1788 > stop VAF = 30%	MYC UP Cell cycle UP	Peri-tumoral TILs	Doc, CP, T, P
BCN1371	HER2-E ER-	Amplified	*PIK3CA* E545K VAF = 73.5%	AR transcription UP ERBB2/3 DOWN MAPK DOWN PI3K DOWN mTOR DOWN	No TILs	Doc, CP, T, P
BCN1369	HER2-E ER-	Amplified		ERBB2/3 UP MAPK UP PI3K UP mTOR UP MUCIN expression UP	No TILs	Pac, T

*Doc* Docetaxel, *CP* Carboplatin, *T* Trastuzumab, *P* Pertuzumab, *Pac* Paclitaxel

### Candidate resistance mechanisms and treatment alternatives

To further explore therapeutic resistance pathways in the proteogenomic data, association analyses between patient-centric RNA, protein and phosphoprotein outliers and the published literature was performed (Supplementary Fig. 12). For each gene or protein, the terms “breast cancer” and “resist OR recur” were used to search for previously studied associations between the outlier genes and breast cancer resistance. Genes with highest filtered PubMed citation counts include well-studied genes such as *ESR1*, *BRCA1/2*, *TP53*, *EGFR* and *AKT1* in addition to *ERBB2* (Supplementary Data 8). As expected, ERBB2 was among the most prominent negative protein and phosphoprotein outliers in BCN1326, BCN1331, and BCN1335 that were associated with the keyword “resistance” (Supplementary Fig. 12). Furthermore, the TOP2A RNA and protein expression also stood out as being strongly associated with “resistance” in BCN1335, the case for whom the amplified locus may to be driving TOP2A rather than ERBB2 expression (Supplementary Fig. 12; Fig. 3d). The most prominent proteomics outlier for patient BCN1369, MUC6, was not associated with citations containing the keyword “resistance” (Supplementary Fig. 12). Nonetheless, multiple mucin family members were outliers with high protein expression specifically in this patient, two of which had citations associated with “resistance” (Supplementary Fig. 12). The consistently high levels of mucin protein expression in patient BCN1369 are clearly discernible in the heatmap shown in Fig. 5e. This observation is notable because mucin expression has been proposed to mask ERBB2 epitopes and prevent trastuzumab binding, as shown previously in cell lines^10,28,29^.

Since therapeutic hypotheses could not be explored directly in non-pCR patients our previously published proteogenomic analysis of a PDX panel was analyzed to determine if any of the molecular features of the resistant tumors in the DP1 study were phenocopied in ERBB2+ patient-derived xenografts (PDX)^6^. Interestingly, WHIM35 has high expression of mucin proteins compared to WHIM8 (Supplementary Fig. 13A) indicating a phenocopy of BCN1369 (Supplementary Fig. 13B). Consistent with cell line-based studies reported in the literature^30–33^ trastuzumab induced tumor regression in mucin negative WHIM8 but not in mucin positive WHIM35 (Fig. 5f). Drawing from the observation that BCN1369 also exhibited elevated PI3K-Akt-mTOR signaling (Fig. 5b), together with a recent report that showed mTOR mediated MUC1 induction in multiple breast cancer cell lines^30^, these PDX models were additionally treated with the small molecule mTOR inhibitor everolimus. Everolimus in combination with ERBB2-targeted therapy induced significant regression (Fig. 5f) in the trastuzumab-resistant WHIM35 model.

## Discussion

Here we report a successful microscaled proteogenomics demonstration project in patients with ERBB2 + breast cancer. Prior efforts at proteomic and phosphoproteomic analysis of needle core biopsies used one-shot analysis^14,34,35^ or off-line SCX fractionation combined with a super-SILAC approach to quantify ~2000–5000 proteins and ~3800 phosphorylation sites per core. None of these prior studies incorporated genomic analyses into their work flows. In contrast, our approach to core biopsy analysis provides deep-scale genomic, proteomic and phosphoproteomic analysis, identifying more  than 25,000 phosphorylation sites in PDX tissue, >17,000 sites in core biopsy tissue, and 11,000 proteins in both for integrative multi-omics analyses.

We illustrate the potential microscaled proteogenomics in a proof-of-principle breast cancer clinical study designed to detect the immediate effects of inhibiting the ERBB2 pathway. Despite a very small cohort size, we were able to detect statistically significant downregulation of ERBB2 protein and phosphosite levels and perturbations in phosphosite signature for downstream mTOR targets specific to samples associated with pCR. Of the 7 (out of 21 total ERBB2 sites identified in Supplementary Data 4) phosphosites from ERBB2 with complete data across the cohort, all showed downregulation in pCR cases (Supplementary Data 5). Of the 21 sites identified, only two have been characterized in detail in cell lines (www.phosphosite.org). These are pY-1248, a known auto-activation site^36^, and pT-701, which may serve as a negative feedback site^37^, although their in-vivo roles are largely unexplored. The role of downregulation of ERBB2 phosphorylation in response to treatment is complicated by the observed downregulation of ERBB2 protein levels, but from a biomarker perspective these are secondary questions that do not negate the primary conclusion that we were able to make a valid pharmacokinetic observation. As important, our ability to resolve complexity in this setting to assess inhibition of ERBB2 signaling is also revealed by downregulation of a signature of target sites for mTOR, a kinase activated downstream of ERBB2, specifically in pCR patients (Fig. 4c).

An initial proteogenomic focus on ERBB2 is readily justified given the biological variability within tumors designated ERBB2 positive. The testing guidelines are designed to offer as many patients anti-ERBB2 treatment as possible, even though it is recognized that this “catch-all” approach likely includes a number of actual negative cases^38^. Our analysis is not intended to be definitive or clinically actionable as the sample size is small and our pipeline is research-based. However, these preliminary analyses suggest three classes of resistance mechanisms to ERBB2-directed therapeutics can be detected. The false ERBB2 + are exemplified by case BCN1326. This case was initially diagnosed by FISH but ERBB2 protein was not over-expressed when re-analyzed using standard IHC (IHC 1+). Three independent pretreatment and three post treatment biopsies were analyzed, which helps rule out tumor heterogeneity as a likely cause of the misdiagnosis. The second class of potential misclassification is pseudo ERBB2+, represented by cases BCN1331 and BCN1335. In these cases, there was evidence for amplification of *ERBB2*, but proteogenomic evidence suggested that ERBB2 is not a strong driver including: (a) lower levels of ERBB2 protein and phosphoprotein compared to pCR cases; (b) low expression from other genes within the minimal ERBB2 amplicon (STARD3, PDAP3 and GRB7); and (c) a paucity of expression of dimerization partners ERBB3 and ERBB4. An orthogonal measurement of ERBB2 levels using single shot parallel reaction monitoring hints at a more efficient approach than the TMT multiplex assay that ultimately could form the basis of a clinical assay (Supplementary Fig. 6). The third resistance class demonstrates lack of pCR despite proteogenomic evidence for true ERBB2 positivity. Here proteogenomic analysis provided candidate mechanisms of resistance to consider, such as the upregulation of mucin proteins, active androgen signaling or the lack of an antitumor immune response.

We emphasize our purpose herein is not to make definitive clinical conclusions, but to illustrate the wide range of resistance biology that microscaled proteogenomics methodologies can reveal, thus promoting further investigation. We acknowledge that the therapeutic alternatives suggested in this pilot study require considerable further study. For example, for BCN1335 the proteogenomic profile (both DNA and protein) suggests that TOP2A is a more likely driver, with higher amplification and protein expression than ERBB2. Other therapeutic hypotheses were raised by evidence for active androgen receptor signaling in BCN1371 (Fig. 5b) and mucin expression in BCN1369 (Fig. 5e). For both examples, there is prior evidence for a role in resistance to trastuzumab but persistent controversy regarding the clinical actionability of these proposed mechanisms^29,39,40^.

The PDX experiments we describe are designed to illustrate how proteogenomic analyses could be used to identify individual PDX that phenocopy hypothetical resistance mechanisms observed in clinical specimens, thus promoting preclinical investigation of alternative treatments that could drive clinical trial design^3,6^. While a clinical trial of everolimus in ERBB2 + breast cancer demonstrated an improvement in progression free survival (PFS) when added to the combination of vinorelbine and trastuzumab but the trial failed because of toxicity. Our results suggest that the use of everolimus in this setting could be reconsidered in ERBB2+ mucin + tumors^41^.

Another important feature of the microscaled proteogenomic analysis presented herein is the ability to assess the immune microenvironment. This is a critical aspect of breast cancer diagnostics with the approval of the PDL1 inhibitor atezolizumab in PDL1 + advanced TNBC^42^. PDL1 IHC is used as a predictive biomarker for atezolizumab, but the optimal approach to the analysis of the immune microenvironment remains under investigation^43^. BCN1326 and BCN1331, examples where the diagnosis of ERBB2 positive status was challenged by proteogenomic analysis, displayed proteomic evidence for PDL1, phospho-PD-L1, and phospho-PD1 expression, consistent with the infiltrating TIL patterns that were observed. Thus, in the future, PD1 and PDL1 assessment by proteogenomics could be considered for prediction of PDL1/PD1 antibody efficacy.

While the microscaled proteogenomic methods are deployed here in the context of a clinical study in breast cancer, they are patently extensible to any other solid tumor. The analyses described are not designed for clinical use, although potentially the time required to go from core needle biopsy to actionable results (2 to 4 weeks) is similar to next generation DNA and RNA sequencing. Analysis time can be reduced with automation of sample processing, the use of faster instrumentation and orthogonal gas phase fraction such as FAIMS^44–46^. Furthermore, the protocol as presented can be readily adapted for use as a diagnostic tool by redirecting some of the denatured protein obtained using the BioTExt procedure to PRM assays developed for targets delineated in larger clinical discovery datasets, as illustrated for ERBB2 (Supplementary Fig. 6).

In conclusion, our study provides the methodology for proteogenomic analysis of core biopsy material from cancer patients. The small cohort size prevents any definitive conclusions regarding the clinical utility; however, we have demonstrated that the identification of relevant proteogenomic features in core biopsies is a feasible exercise. We can now seek definitive clinical conclusions through analyses involving larger numbers of patients.

## Methods

### Patient-derived xenografts and drug treatment

For PDX studies, all animal procedures were approved by the Institutional Animal Care and Use Committee at Baylor College of Medicine (Houston, TX, USA) (protocol# AN-6934). 2–3 mm tumor pieces from PDX tumors were engrafted into cleared mammary fat pads of 3–4 weeks old SCID/bg mice (Envigo) and allowed to grow without exogenous estrogen supplementation until tumors reached 200–250 mm^3^. The human tissue used for PDX generation was collected at Washington University St Louis and with appropriate patient consenting. For the core and bulk comparison experiment, two non-adjacent cores were first obtained from the PDX models, immediately embedded in optimal cutting temperature (OCT) medium and snap-frozen in liquid nitrogen. Following coring, tumors were surgically resected, and the tumor bulk were snap-frozen in liquid nitrogen. For treatment experiments, mice were randomized into 4 groups receiving (i) vehicle or control; (ii) everolimus (5 mg per kilogram (kg) body weight in chow daily); (iii) trastuzumab (30 mg per kg body weight weekly by intraperitoneal injection) or iv) a combination of trastuzumab and everolimus (administered as described in (ii) and (iii)). There were *n* = 15 mice per arm. Tumor volumes were measured by caliper every 3–4 days. For all animal experiments, tumor volumes were calculated by $V = \frac{4}{3} \ast \pi \ast \left( {\frac{{{\mathrm{Length}}}}{2}} \right)^2 \ast \left( {\frac{{{\mathrm{Width}}}}{2}} \right)$. Baseline samples were collected on the day of randomization and treatment start date followed by sample collection at 1-week and 4-week post-treatment. Animals were sacrificed when tumors reached 1500 mm^3^ or at the study end time-point.

### DP1 Clinical Data

Following informed consent, patients diagnosed ERBB2 positive via diagnostic breast biopsy were enrolled in the National Surgical Adjuvant Breast and Bowel Project (NSABP) Biospecimen Discovery Project (DP1) for ERBB2 + breast cancer (https://clinicaltrials.gov/ct2/show/study/NCT01850628). In accordance with consent, regular cancer care and optional additional 14-gauge needle biopsies preserved in optimal cutting temperature (OCT) fixative were collected at diagnostic breast biopsy and 48 to 72 h following chemotherapy and anti-ERBB2 therapy. Blood samples were also collected and compacted to a frozen pellet before the start of standard treatment, up to 3 weeks after the first dose but before the second dose, and at the time of surgery and sent to Washington University (St. Louis, MO) for research purposes.

Biopsy samples, blood samples, and medical information (including pathology reports) were collected and labeled with a study number, which was a unique code assigned to samples and medical information. This unique code number, which is linked to the patient’s name, was kept separate from other sample information. Sample was given a separate unique BCN number for each patient (i.e., BCN“XXXX”) upon enrollment in the study. All subsequent sample derivatives were associated with the corresponding BCN number.

Patients were able to withdraw samples without any penalty or loss of benefits entitled. However, in order to protect the anonymity of the databases, DNA sequences or other information that came from samples were not removed once entered into databases to prevent the risk of identification.

### Biopsy Trifecta Extraction embedding and sectioning

14-gauge needle human biopsies were embedded in OCT fixative and stored at −80 °C. Utilizing a cryostat maintained between −15 to −23 °C, each biopsy was sectioned at 50 microns. Six (6) 50-micron curls were alternated amongst three (3) 1.5 mL microcentrifuge tubes assigned for denatured protein-DNA, native protein-DNA, or RNA extraction. At the start of sectioning and after an interval of six (6) curls were sectioned, a 5 micron curl was mounted on a slide for Hematoxylin and Eosin (H&E) staining and histopathological confirmation. This process was repeated until six (6) 50-micron curls were collected in all tubes per sample. The samples were then shipped from Washington University (St. Louis, MO) to Baylor College of Medicine (Houston, TX) for subsequent processing. The tumor content percentages of each biopsy H&E slide (TC1, TC2, TC3, and TC4) were recorded and calculated to form a mean tumor content (avgTC) for that biopsy. Those biopsies with an avgTC less than 50% were removed from further processing.

### Immunohistochemistry

Tissue sections on charged glass slides were cut to 5 µm and deparaffinised in xylene and rehydrated via an ethanol step gradient. Peroxidase blocking, heat-induced antigen retrieval, and primary antibody incubation were performed per standard protocol under the following abbreviated conditions: ERBB2 (SP3, Neomarkers) 1:100, Tris pH 9.0; AR (441, sc-7305, Santa Cruz) 1:50, Tris pH 9.0; Muc1 (sc-7313, Santa Cruz) 1:150, Citrate pH 6.0; CD3 (polyclonal, A0452, Dako) 1:100, Tris pH 9.0. All primary antibodies were incubated at room temperature for 1 h followed by standard chromogenic staining with the Envision Polymer-HRP anti-mouse/3,3′diaminobenzidine (DAB; Dako) process. Immunohistochemistry scoring were performed using established guidelines, when appropriate. All IHC results were evaluated against positive and negative controls.

### BioTExt denatured protein extraction

1 mL of cold 70% ethanol (EtOH) was added to tubes assigned for denatured protein. Each tube was quickly pulse-vortexed for 30 s and briefly centrifuged at 20,000 x g for 5 min at 4 °C. The 70% EtOH was carefully aspirated. 1 mL of cold NanoPure water was added, and the tube was quickly pulse-vortexed for 30 s and briefly centrifuged at 20,000 x g for 5 min at 4 °C. The NanoPure water was carefully aspirated. 1 mL of cold 100% EtOH was added, and the tube was quickly pulse-vortexed for 30 s and briefly centrifuged at 20,000 x g for 5 min at 4 °C. The 100% EtOH was carefully aspirated. 100 µL of denatured protein lysis buffer (8 M urea, 75 mM NaCl, 1 mM EDTA, 50 mM Tris-Cl pH 8.0, 10 mM NaF, Phosphatase inhibitor cocktail 2 (Sigma; P5726), Phosphatase inhibitor cocktail 3 (Sigma; P0044), Aprotinin (Sigma; A6103), Leupeptin (Roche; 11017101001), PMSF (Sigma; 78830)) was added to each sample, which was then transferred to a micro-sonicator vial. All samples were incubated on ice for 10 min. Following incubation, samples were individually sonicated in the S220 Ultrasonicator for 2 min at peak power: 100.0, duty factor: 10.0, cycles per burst: 500. Lysates were transferred to 1.5 mL labeled tubes and centrifuged at 4 °C, maximum speed (20,000x*g*), for 30 min. Lysate supernatants containing denatured proteins were transferred to a new labeled tube. The remaining precipitated pellets were snap frozen for subsequent DNA isolation. Quality control of the denatured protein was validated via mass spectrophotometer analysis.

### BioTExt DNA extraction

DNA was isolated via QIAamp DNA Mini Kit (Qiagen; 51306). DNA pellets were equilibrated to room temperature. 100 µL of Buffer ATL and then 20 µL of proteinase K were added to each sample and mixed by vortex. Samples were then incubated at 56 °C for 3 h in a shaking heat block. Following incubation, samples were briefly centrifuged. 20 µL of RNase A (20 mg per mL) was added to each sample, which was then pulse-vortexed for 15 s and incubated for 2 min at room temperature. Samples were briefly centrifuged then pulse-vortexed for 15 s and incubated at 70 °C for 10 min. Following a brief centrifugation of the sample, 200 µL of Buffer AL was added to the sample, which was then pulse-vortexed for 15 s and incubated for an additional 70 °C for 10 min. Following another brief centrifugation, samples were carefully applied to a corresponding QIAamp Mini spin column placed in a collection tube without wetting the rim. The spin columns with sample were centrifuged at 6000x*g* for 1 min and then placed in a new collection tube while discarding the original filtrate. 500 µL of Buffer AW2 was added to spin columns without wetting the rim. Spin columns were centrifuged at maximum speed (20,000x*g*) for 3 min. Following centrifugation, the spin columns were placed in new collection tubes and once again centrifuged at maximum speed for 1 min. Spin columns were then placed in new 1.5 mL micro-centrifuge tubes. 100 µL of Buffer AE was added to each spin column and incubated at room temperature for 5 min while in a shaking heat block. The final DNA isolates were collected in their corresponding 1.5 mL tubes following centrifugation at 6000x*g* for 1 min. DNA quality control was validated via Picogreen analysis.

### BioTExt RNA extraction

1 mL of TRIzol Reagent (Thermo Fisher Scientific; 15596026) was added to each RNA-designated tube of cryo-sectioned curls, which was immediately inverted three times followed by transfer of its contents to a sonicator vial. Samples were individually sonicated in the S220 Ultrasonicator for 2 min at peak power: 100.0, duty factor: 10.0, cycles/burst: 500. All samples were then incubated for 5 min and then transferred to 1.5 mL microcentrifuge tubes. Following addition of 200 µL of chloroform, each sample was incubated for 3 min and then centrifuged at 12,000x*g* for 15 min at 4 °C. The supernatants were discarded. The pellet was air dried in the micro-centrifuge tube for 10 min. The pellet was re-suspended in 20 µL of RNase-free water and incubated at 56–60 °C in a heat block for 10–15 min. RNA was isolated using RNeasy Mini kit (Qiagen; 74106). 10 µL of Buffer RDD and 2.5 µL of DNase I (Qiagen; 79254) was added to each sample. The sample volume was then brought up to 100 µL with RNase-free water, and the sample incubated at room temperature for 10 min. 350 µL of Buffer RLT was added and mixed well with each sample. Thereafter, 250 µL of 100 % EtOH was mixed with each sample, and the mixture was quickly transferred to an RNeasy MinElute spin column (Qiagen; 74106) and placed in a 2 mL collection tube, which was then centrifuged at 12,000x*g* for 15 s. The flow through was discarded, and 500 µL of 80% EtOH was added to each spin column. The columns were centrifuged at 12,000x*g* for 2 min. The flow through was discarded and the column in placed in a new 2 mL collection tube. The samples were centrifuged at full speed for 5 min with the lid of the spin column open. Following centrifugation, the spin column was placed in a 1.5 mL micro-centrifuge tube, and 14 µL of RNase-free water was directly added to the center of the spin column membrane. The spin columns were centrifuged at max speed for 1 min to elute the RNA. RNA quality control was validated via Picogreen analysis.

### BioTExt native protein extraction

100 µL of native protein lysis buffer (50 mM HEPES pH 7.5, 150 mM NaCl, 0.5% Triton X-100, 1 mM EDTA, 1 mM EGTA, 10 mM NaF, 2.5 mM NaVO_4_, Protease inhibitor cocktail, Phosphatase inhibitor cocktail) was added to each native protein sample, which was then transferred to a micro-sonicator vial. Each lysate tube was assigned a trackable Mass Spectrometer label. Lysate concentration measured via Bradford assay of 10 µL of each sample mixed with 800 µL of deionized water. 200 µL of Bradford reagent was added to each deionized water plus lysate aliquot. Each sample was inverted and transferred to assigned cuvettes. Lysates were measured via a spectrophotometer with a corresponding blank sample.

### Genomic data generation and QC analysis

DNA from core biopsies and germline blood samples was PicoGreen quantified. Samples that met the minimum PicoGreen quantified input requirements (≥300 ng DNA, preferred concentration 10 ng/μL) proceeded into the Somatic Whole Exome workflow by which DNA was processed for Somatic Whole Exome Sequencing. This process included library preparation, hybrid capture, sequencing with 76 bp paired-end reads, sample identification QC check, and product-utilized ligation-based library preparation followed by hybrid capture with the Illumina Rapid Capture Exome enrichment kit with 38 Mb target territory.

All libraries were sequenced to attempt to meet a goal of 85% of targets covered at greater than 50x coverage (+/− 5%) for tumor samples utilizing the Laboratory Picard bioinformatics pipeline. All sequencing was performed by the Laboratory on Illumina instruments with 76 base pair, paired‐end sequencing. The Laboratory Picard pipeline aggregated all data from a particular sample into a single BAM file that included all reads, all bases from all reads, and original/vendor-assigned quality scores.

DNA samples were additionally processed for Fluidigm Fingerprint Checks. By genotyping a panel of highly polymorphic SNPs (including SNPs on chromosomes X and Y), a unique genetic ‘fingerprint’ is generated for each sample. These genotypes are stored in the sample tracking database and compared automatically to genotypes from the production pipeline to ensure the integrity of sample tracking.

### Identification of mutations by whole exome sequencing

VarScan2 was used to identify germline mutations (SNPs and INDELs) from the germline BAM files and somatic mutations by comparing the tumor BAM file to the germline BAM file for each patient. Annovar was then used to separately annotate SNP and INDEL vcf files from VarScan for germline and somatic mutations from each patient. Mutations with “non-synonymous SNVs”, “stopgain”, “stoploss”, and “splicing” annotations that affect the protein coding sequences of genes were extracted from the resulting SNP multianno files and combined into a single text file for all patients. Similarly, INDELs annotated as occurring in the exons of genes were extracted from each INDEL multianno file and combined into a single file. The somatic SNPs and INDELs were combined into a single mutation by patient table (unique mutations; Supplementary Data 3A) and a single mutated gene by patient table (Supplementary Data 3B).

### Analysis of copy number alterations

We used the R Package CopywriteR (version 1.18.0)^47^, which uses off-target wholes exome sequencing (WXS) reads, to infer copy number values. All the tumor and normal WXS data had sufficient (>5 million) off-target reads for SCNA detection as recommended by the software. The circular binary segmentation (CBS) algorithm implemented in the CopywriteR package was used for copy number segmentation with the default parameters. From the segmentation results, we use a straightforward weighted-sum approach to summarize the chromosome instability for each sample^5^. Specifically, the absolute log2 ratios of all segments (indicating the copy number aberration of these segments) within a chromosome were weighted by the segment length and summed up to derive the instability score for the chromosome. The genome-wide chromosome instability index was derived by summing up the instability score of all 22 autosomes. Next, we used GISTIC2 (version 2.0.23^48^) to retrieve gene-level copy number values (Supplementary Data 3C) and call significant copy number alterations in the cohort (integer calls). The stringent threshold (2 or −2) of the integer call results were used to define genes with copy number aberration.

### RNA-sequencing data generation and analysis

RNA was quantified via RiboGreen, and RNA quality was measured by the RQS (RNA Quality Score). Samples that did not meet the minimum RiboGreen quantified input requirements (≥500 ng RNA, preferred concentration 10 ng/μL, RQS > 5.5) were held for further evaluation. RNA samples of sufficient quality were processed for Long-Insert Strand-Specific Transcriptome Sequencing. Library preparation utilizes a unique high-quality, high-throughput, low-input process using the Illumina TruSeq RNA protocol, which generates poly-A mRNA libraries from total RNA using oligo dT beads. The RNA sequencing library construction includes poly-A selection, cDNA synthesis and library construction using the strand specific Illumina TruSeq Protocol. Each RNA sample entering library construction receives an aliquot of (ThermoFisher) ERCC Controls. All libraries are sequenced to attempt to meet a goal of 50 M reads aligned in pairs (+/− 5%) at 101 bp read length using the Illumina platform as measured using our Picard bioinformatics pipeline. The Picard pipeline aggregates all data from a particular sample into a single demultiplexed, aligned BAM file which includes all reads, all bases from all reads and original/vendor assigned quality scores.

The SamToFastq function from Picard tools was used to convert the BAM file to fastq files for each sample. The RSEM tool was used to calculate both estimated read counts (RSEM) and Fragments Per Kilobase of transcript per Million mapped reads (FPKM) for each gene from the fastq files^49^. For the analyses described in the manuscript, non-0 RSEM data for each sample was upper-quartile normalized, and genes with 0 read counts across all samples were removed (0 reads treated as NA). ESTIMATE, Cibersort, and xCell were used to infer stroma content (ESTIMATE and xCell), immune infiltration (all), and tumor immune cell profiles (Cibersort and xCell) using upper quartile normalized RSEM data, and log2 transformed upper quartile normalized RSEM data was used for the outlier and LIMMA analyses described below^50-52^.

### Experimental design for MiProt

For the CPTAC workflow, the 4 PDX models were analyzed in process replicates (8 TMT channels) along with 2 common reference (CR) samples in a TMT ten-plex format. The first common reference (CR1) was constructed from equal proportions of peptides derived from the 4 cryopulverized PDX bulk tumors. The second common reference (CR2) had been used in a prior proteogenomic breast cancer PDX study that included these four models^6^. For the MiProt workflow, 8 individual cores comprising 2 cores per model (8 TMT channels) were analyzed along with 2 common references in TMT ten-plex format. The first CR (CR3) was composed of equal proportions of peptides from the 8 cores, while the second CR was an aliquot of the bulk CR (CR1), described above. Protein and phosphosite expression were reported as the log ratio of each sample’s TMT intensity to the intensity of an internal common reference includes in each plex, either CR1 for the CPTAC workflow or CR3 for the MiProt workflow. For analyzing patient derived core needle biopsies, the TMT-eleven-plex format were used, where the first 9 channels contained peptides from 9 core needle biopsies and the last two channels (131 N, 131 C) contains two different CRs. Channel 131 N contained CR4 that was constructed from equal proportion of peptides from all the 14 patients. Channel 131 C contained CR5 that has been previously used to characterize a large cohort of breast cancer subtypes (https://cptac-data-portal.georgetown.edu/cptac/study/disclaimer?accNum=S039). For this manuscript, all ratios were calculated relative to CR4. For both PDX and clinical core analyses, samples within a TMT11 plex were randomized to reduce batch effects (Supplementary Data 1).

### Proteomic sample preparation for MiProt analysis

Protein lysates in 8 M Urea were treated with 1 mM DTT for 45 min followed by 2 mM iodoacetamide (IAA) for an additional 45 min. 8 M Urea was diluted to a final concentration of 2 M with 50 mM Tris-HCL pH 8.5. Protein lysates were incubated with endopeptidase LysC (Promega) at a concentration of 1:50 (μg of LysC to μg of Proteins) for 2 h followed by overnight incubation with Trypsin (Promega) at a concentration of 1:30 (μg of Trypsin to μg of Proteins). Both enzymatic digestions were performed at room-temperature. Following protein digestion, peptides were acidified to a final concentration of 1% Formic acid followed by purification using 50 mg Sep-Pak cartridge (Waters). Peptides were eluted off the Sep-Pak cartridge with 50% acetonitrile and 0.1% formic acid. Peptide concentration was measured using 280 absorbance using a Nanodrop (Thermo Scientific). For qualitative assessment, 0.5 μg peptides were run on a nLC1200 coupled to Q-Exactive + LC-MS setup (Thermo Scientific). Eluted peptides were snap-frozen and dried using a speed-vac apparatus. For the CPTAC workflow, a total of 300 μg peptides were labeled with 800 μg TMT reagent as described previosly^8^. For the MiProt workflow a total of 25 μg peptides in 100 μL of 50 mM Hepes pH8.5 was labeled with 200 μg TMT reagent (8-fold excess). TMT and peptide mixture were incubated at room-temperature for 1 h. Prior to the quenching of excess TMT reagent, a total of 1 μL per sample was stage-tipped onto a C18 disc (EMPORE C18) and a total of 0.5 μg of peptides was run on a 30 min gradient to assess TMT labeling efficiency. In addition, 2 μL from each sample were pooled together and desalted. A total of 0.5 μg peptides were run on a 110 min gradient to assess mixing ratios. We allowed partial TMT labeling to be over 99%, fully TMT labeling to be over 94% and mixing ratios to be within +/− 15% compared to the common internal reference (CR), which was 131 N for TMT10-plex setup and 131 C for TMT11-plex setup. Excess TMT reagent was quenched using 6 μL of 5% Hydroxylamine (Sigma) followed by a 15 min incubation. All the samples within a plex were mixed based on the mixing ratios to achieve equal amounts for all channels. Peptides were purified using a 100 mg Sep-Pak cartidge (Waters) and dried down using a speed-vac apparatus.

### Basic reverse fractionation and phosphoenrichment

For basic phase reverse (bRP) fractionation, ~250 μg of peptides were dissolved in 500 μL of 5 mM ammonium formate and 5% acetonitrile. An offline Agilent 1260 LC coupled to 30 cm and 2.1 diameter column running at a flow-rate of 200 μL per minute was used for bRP fractionation. Peptides were fractionated into 72 fractions and finally concatenated into 24 fractions. A total of 2 μg peptides per fraction was transferred into the mass-spectrometer vial for whole proteome analysis, but only 0.5 μg per fraction was injected for whole proteome analysis. The 24 fractions were further concatenated (by pooling of every 6th fraction) into 4 fractions (~62 μg peptides per fraction) for phosphopeptide enrichment.

The CPTAC workflow has been described before^2^. In brief, for the CPTAC workflow, 3000 μg of peptides were dissolved in 1000 μL of 5 mM ammonium formate and 5% acetonitrile. Offline fractionation was performed as described above using a 30 cm and 4.6 diameter column. A total of 72 fractions were concatenated into a total of 24 fractions and 0.5 μg peptides per fraction were analyzed for whole cell proteomics. The 24 fractions were further concatenated into a total of 12 fractions (by pooling of every 2nd fraction yielding ~250 μg per fraction) for IMAC based phosphopeptide enrichment.

Phosphopeptide enrichment was done using Fe3 + immobilized metal affinity chromatography (IMAC). For this, Ni-NTA (Qiagen) beads were washed three times with HPLC grade water followed by incubation with 100 mM EDTA (Sigma) for 30 min to strip Ni^2+^ off the beads. The beads were washed 3 times with HPLC grade water followed by incubation with FeCl_3_ (Sigma) for 45 min. Beads were again washed with HPLC grade water followed by resuspension of Fe_3_^+^ loaded agarose beads with resuspension buffer containing methanol, acetonitrile and 0.01% acetic acid at 1:1:1 ratio. For both CPTAC and MiProt workflows, dried down peptides were resuspended to a final volume of 500 μL in 50% acetonitrile and 0.1% trifluoroacetic acid (TFA) and supplemented with 97% acetonitrile and 0.1% TFA to a final concentration of 80% acetonitrile and 0.1% TFA. A total of 20 μL of 50% slurry was used per fraction for phosphopeptide enrichment. IMAC beads and peptides were incubated at room temperature for 30 min on a tumble-top rotator. Beads were spun down and resuspended with 200 μL of 80% acetonitrile and 0.1% TFA and transferred directly onto a conditioned C18 stage-tips. Phosphopeptides were eluted off the beads using 500 mM K_2_HPO_4_, pH 7 buffer onto C18 stage-tip, washed with 1% formic acid and finally eluted into a mass spectrometer LC vial using 50% acetonitrile and 0.1% FA.

### Proteomic data acquisition and processing

A Proxeon nLC-1200 coupled to Thermo Lumos instrumentation was used for proteome and phosphoproteome data acquisition. Peptides were run on a 110 min gradient with 86 min of effective gradient (6 to 30% buffer B containing 90% ACN and 0.1%FA). For phosphoproteomics analysis of cores, a second injection was performed and analyzed over a 145 min gradient with 120 min of effective gradient (6 to 30% buffer B containing 90% ACN and 0.1% FA). The acquisition parameters are as follows, MS1: resolution- 60,000, MS1 injection time: 50 s, MS2: resolution: 50,000, MS2 injection time: 110 s, AGC 5E4. Data acquisition was performed with a cycle time of 2 s.

Raw files were searched against the human (clinical samples) or human and mouse (PDX samples) RefSeq protein databases complemented with 553 small-open reading frames (smORFs) and common contaminants (Human: RefSeq.20111003_Human_ucsc_hg38_cpdb_mito_259contamsnr_553smORFS), (Human and Mouse:RefSeq.20160914_Human_Mouse_ucsc_hg19_mm10_customProDBnr_mito_150contams) using Spectrum Mill suite vB.06.01.202 (Broad Institute and Agilent Technologies) as previously described in detail^6^. For TMT quantification, the ‘Full, Lys only’ option that requires lysine to be fully labeled while allowing under-labeling of peptides *N*-termini was used. Carbamidomethylation of cysteines was set as a fixed modification, and N-terminal protein acetylation, oxidation of methionine (Met-ox), de-amidation of asparagine, and cyclization of peptide N-terminal glutamine and carbamidomethylated cysteine to pyroglutamic acid (pyroGlu) and pyro-carbamidomethyl cysteine were set as variable modifications. For phosphoproteome analysis, phosphorylation of serine, threonine, and tyrosine were allowed as additional variable modifications, while de-amidation of asparagine was disabled. Trypsin Allow P was specified as the proteolytic enzyme with up to 4 missed cleavage sites allowed. For proteome analysis, the allowed precursor mass shift range was −18 to 64 Da to allow for pyroGlu and up to 4 Met-ox per peptide. For phosphoproteome analysis, the range was expanded to −18 to 272 Da, to allow for up to 3 phosphorylations and 2 Met-ox per peptide. Precursor and product mass tolerances were set to ±20 ppm and peptide FDR to 1 % employing a target-decoy approach using reversed protein sequences^42^. For PDX analyses, the subgroup-specific (SGS) option in Spectrum Mill was enabled as previously described^6^.This allowed us to better dissect proteins of human and mouse origin. If specific evidence for BOTH human and mouse peptides from an orthologous protein were observed, then peptides that cannot distinguish the two (shared) were ignored. However, the peptides shared between species were retained if there was specific evidence for only one of the species, thus yielding a protein group with a single subgroup attributed to only the single species consistent with the specific peptides.

For generation of protein and phosphopeptide ratios, reporter ion signals were corrected for isotope impurities and relative abundances of proteins, and phosphorylation sites were determined using the median of TMT reporter ion intensity ratios from all PSMs matching to the protein or phosphorylation site. PSMs lacking a TMT label, having a precursor ion purity <50%, or having a negative delta forward-reverse score (half of all false-positive identifications) were excluded. To normalize quantitative data across TMT10/11plex experiments, TMT intensities were divided by the specified common reference for each phosphosite and protein. Log2 TMT rations were further normalized by median centering and median absolute deviation scaling.

### Parallel reaction monitoring

Two unique peptides for ERBB2 protein (VLQGLPR and GLQSLPTHDPSPLQR) were used for PRM analysis. Peptides used for proteome analysis were analyzed by Orbitrap Fusion Lumos mass spectrometer coupled with the EASY-nLC1200 system (Thermo Fisher Scientific) for PRM analysis. 1 μg of peptides was loaded to a trap column (150 μm × 2 cm, particle size 1.9 μm) with a max pressure of 280 bar using Solvent A (0.1% formic acid in water) and then separated on a silica microcolumn (150 μm × 5 cm, particle size, 1.9 μm) with a gradient of 4–28% mobile phase B (90% acetonitrile and 0.1% formic acid) at a flow rate of 750 nl per min for 75 min. Both data-dependent acquisition (DDA) and PRM modes were used in parallel. For DDA scans, a precursor scan was performed in the Orbitrap by scanning *m/z* 300–1200 with a resolution of 120,000 at 200 *m/z*. The most 20 intense ions were isolated by Quadrupole with a 2 *m/z* window and fragmented by higher energy collisional dissociation (HCD) with normalized collision energy of 32% and detected by ion trap with rapid scan rate. Automatic gain control targets were 5 × 10^5^ ions with a maximum injection time of 50 ms for precursor scans and 10^4^ with a maximum injection time of 50 ms for MS2 scans. Dynamic exclusion time was 20 s (±7 ppm). For PRM scans, pre-selected peptides were isolated by quadrupole with a 0.7 *m/z* window followed by HCD with normalized collision energy of 32%, and product ions (MS2) were scanned by Orbitrap with a resolution of 30,000 at 200 *m/z*. Scan windows were set to 4 min for each peptide. For relative quantification, the raw spectrum file was crunched to mgf format by Proteome Discoverer 2.0 software (Thermo Fisher Scientific) and then imported to Skyline along with the raw data file. We validated each result by deleting non-identified spectra and adjusting the AUC range. Finally, the sum of the area of at least six strongest product ions for each peptide was used for the result.

### Network-based gene function prediction

Co-expression network construction using mRNA and protein expression data and network-based gene function prediction for KEGG pathways were performed^21^ and implemented in OmicsEV (https://github.com/bzhanglab/OmicsEV).

### Outlier analysis

The data for each gene or protein from the set of baseline samples from the patients that showed pathological complete response was used to establish a normal distribution for that gene/protein. For each gene, a *Z*-score for each baseline sample from the non-pCR case was calculated by determining the number of standard deviations the expression value in the non-pCR deviated from the mean of this distribution. Genes/proteins with low variance (variance < 1.5) and that did not have a normal distribution, by Shapiro-Wilk test, in the patients showing pathological complete response were removed prior to subsequent analysis. For phosphoprotein level outlier analysis, the mean level of all phosphosites for each protein was evaluated. Pathway analysis using single sample Gene Set Enrichment Analysis (ssGSEA) and the MSigDb gene sets was carried out on the outlier *Z*-scores for each dataset in each non-pCR sample using the parameters described below.

### Differential analysis using limma

The limma R package was used to analyze the set of patients with both on-treatment and pre-treatment cores in order to compare on-treatment vs. pre-treatment expression in pCR and non-pCR patients separately in each dataset (RNA, protein, phosphoprotein (mean phosphosite level for each protein), and phosphosite datasets) and to compare on-treatment vs. pre-treatment changes in expression in pCR patients to non-pCR patients. Samples from BCN1368 and BCN1369 were excluded from this analysis because of they did not receive the full treatment regimen (didn’t get pertuzumab). Phosphosite level data for this analysis was first processed by taking the mean of all peptides containing each fully localized site as determined by Spectrum Mill. For this analysis, duplicate cores for a given patient were included but the limma duplicateCorrelation function was used to derive a consensus for each patient for the differential analysis. Each gene (or site) in each dataset was fitted to a linear model with coefficients for each group (on-treatment pCR, pre-treatment pCR, on-treatment non-pCR, and pre-treatment non-PCR) and each plex (to account for batch effects), and moderated *T*-tests for each comparison were carried out by limma using the residual variances estimated from the linear models. PTM-SEA was applied to signed, log10 transformed *p*-values from this analysis using the parameters described below.

### Geneset enrichment and PTM-signature enrichment analyses

Pathway analysis was performed using single sample Gene Set Enrichment Analysis (ssGSEA) and post-translational modification signature enrichment analysis (PTM-SEA). Protein and phosphosite measurements of technical replicates were combined by taking the average across replicates before subsequent analysis. Pathway level comparisons of bulk and core material were based on signed, log10-transformed *p*-values derived from a moderated two-sample *T*-test using the limma R-package comparing luminal and basal tumors separately for bulk and core samples. For proteome data we first applied the two-sample moderated *T*-test for each protein and resulting transformed *p*-values (see above) were collapsed to gene-centric level for ssGSEA by retaining the most significant *p*-value per gene symbol. Phosphosite-level data were subjected to limma-analysis to derive transformed *p*-values (see above) for each phosphorylation site. Sequence windows flanking the phosphorylation site by 7 amino acids in both directions were used as unique site identifier. For PTM-SEA, only fully localized phosphorylation sites as determined by Spectrum Mill software were taking into consideration. Phosphorylation sites on multiply phosphorylated peptides were resolved using methods described in Krug et al^15^.

### Pubmed crawling

An in-house Python script was used to drive queries using NCBI-s E-utilities, and resulting freely available information (title, abstract, keywords) were saved to a local SQL database. For each publication, a case-insensitive text search for “resist” OR “recur” AND “breast cancer” was performed, with positive hits retained and tallied for each gene. Publications with over 100 different gene associations were excluded to avoid false positives from high-throughput studies. The results are available in Supplementary Data 8.

### Additional statistical analyses and R code

*T*-tests, Shapiro-Wilk tests and Wilcoxon rank sum and signed rank tests were performed using base R (http://www.R-project.org/). Spearman correlation analyses were performed using the R Hmisc package (https://CRAN.R-project.org/package=Hmisc). Heatmaps were generated using the heatmap.2 function in the gplots R package (https://CRAN.R-project.org/package=gplots) and Morpheus (https://github.com/cmap/morpheus.R) and R code will be made available upon request.

### Reporting summary

Further information on research design is available in the Nature Research Reporting Summary linked to this article.

## Supplementary information

Supplementary Information

Reporting Summary

Description of Additional Supplementary Files

Supplementary Data 1

Supplementary Data 2

Supplementary Data 3

Supplementary Data 4

Supplementary Data 5

Supplementary Data 6

Supplementary Data 7

Supplementary Data 8

## Data Availability

The genomics data have been deposited in the dbGAP database under the accession code phs001907.v1.p1. The proteomics data used in this study is available CPTAC portal at the link https://cptac-data-portal.georgetown.edu/cptac/s/S051. The source data underlying all main figures and Supplementary Figs. are provided as a Source Data file 1–8 and is appropriately cited in the result section. A reporting summary for this article is available as a Supplementary Information file.

## Code availability

All codes used in this study are published and are appropriately cited throughout the text. The code used for outlier analysis will be available upon request.
