# Supplementary material for: Microscaled proteogenomic methods for precision oncology
Source: Nat Commun. 2020 Jan 27;11:532. doi: 10.1038/s41467-020-14381-2 (PMC6985126; doi:10.1038/s41467-020-14381-2)
Supplement: Supplementary file 1 — Supplementary Information [file 41467_2020_14381_MOESM1_ESM.pdf]

Supplementary Information

Microscaled Proteogenomic Methods for Precision Oncology

Shankha Satpathy[1*#], Eric J. Jaehnig[2*], Karsten Krug[1], Beom-Jun Kim[2], Alexander B. Saltzman[3], Doug W. Chan[2], Kimberly R. Holloway[2], Meenakshi Anurag[2], Chen Huang[2], Purba Singh[2], Ari Gao[2], Noel Namai[2], Yongchao Dou[2], Bo Wen[2], Suhas Vasaikar[2], David Mutch[4], Mark A. Watson[4], Cynthia Ma[4], Foluso O. Ademuyiwa[4], Mothaffar Rimawi[2], Rachel Schiff[2], Jeremy Hoog[4], Samuel Jacobs[5], Anna Malovannaya[3], Terry Hyslop[6], Karl C. Clauser[1], D. R. Mani[1], Charles Perou[7], George Miles[2], Bing Zhang[2], Michael A. Gillette[1,8], Steven A. Carr[1#], Matthew J. Ellis[2#]

[1]Broad Institute of Harvard and Massachusetts Institute of Technology, Cambridge, Massachusetts MA 02142, USA
[2]Lester and Sue Smith Breast Center and Dan L Duncan Comprehensive Cancer Center, Baylor College of Medicine, Houston, TX 77030, USA
[3]Verna and Marrs McLean Department of Biochemistry and Molecular Biology, Baylor College of Medicine, Houston, TX 77030, USA
[4]Siteman Comprehensive Cancer Center and Washington University School of Medicine, St. Louis, MO 63110, USA
[5]NSABP Foundation, Pittsburgh, PA 15212, USA
[6]Department of Biostatistics and Bioinformatics, Duke University Medical Center, Durham, NC 27710, USA
[7]Lineberger Comprehensive Cancer Center, University of North Carolina at Chapel Hill, Chapel Hill, NC 27514, USA
[8]Division of Pulmonary and Critical Care Medicine, Massachusetts General Hospital, Boston, MA 02115, USA

*These authors contributed equally.

#corresponding authors SS: shankha@broadinstute.org SAC: scarr@broad.mit.edu and MJE: Matthew.Ellis@bcm.edu

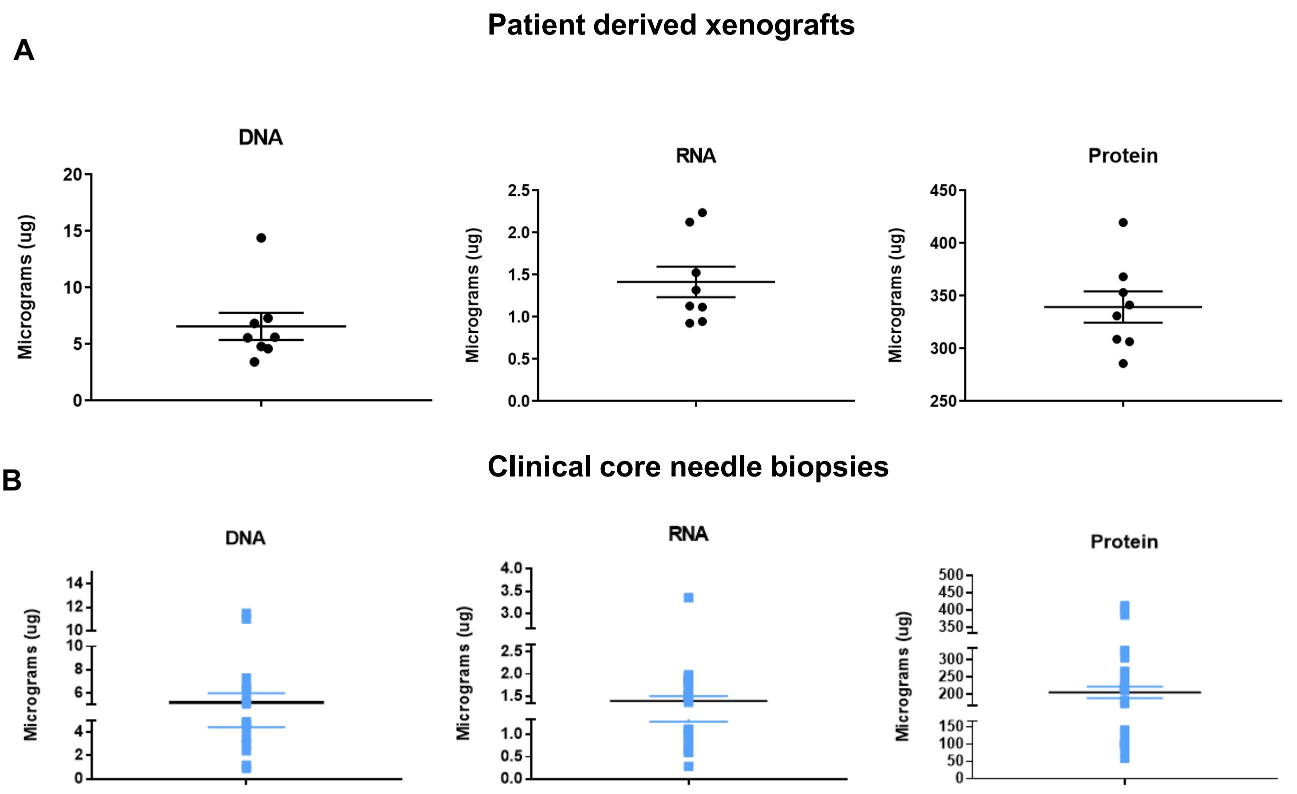

Supplementary Figure 1. DNA, RNA and protein yields from core needle biopsies processed using BioTExt. **A**. Box plot showing DNA, RNA and Protein yields from a total of 8 core needle biopsies from 4 PDX Models: WHIM4, 14, 18 and 20. Error bars represent standard error of mean (SEM).
B. Box and scatter plots showing DNA, RNA and protein yields from all core needle biopsies that were processed from the DP1 study. Samples with no yield were excluded. Error bars represent standard error of mean (SEM).

A

PDX model	WHIM18	WHIM18	WHIM20	WHIM20	WHIM14	WHIM14	WHIM2	WHIM2
Experiment	Bulk	Bulk	Bulk	Bulk	Bulk	Bulk	Bulk	Bulk
BRCA subtype	Luminal	Luminal	Luminal	Luminal	Basal	Basal	Basal	Basal
Replicates	1	2	1	2	1	2	1	2
Number of quantified proteins	12196	12196	12192	12196	12192	12195	12194	12194
Number of quantified phosphosites	41842	41820	41813	41823	41820	41828	41841	41812

PDX model	WHIM18	WHIM18	WHIM20	WHIM20	WHIM14	WHIM14	WHIM2	WHIM2
Experiment	Core	Core	Core	Core	Core	Core	Core	Core
BRCA subtype	Luminal	Luminal	Luminal	Luminal	Basal	Basal	Basal	Basal
Replicates	1	2	1	2	1	2	1	2
No	11314	11315	11316	11317	11317	11317	11317	11315
Number of phosphosites	25788	25791	25785	25778	25781	25786	25787	25780

B

PDX model	Proteome	Phosphoproteome
	Bulk	Bulk
WHIM18	0.95	0.91
WHIM20	0.93	0.89
WHIM14	0.95	0.91
WHIM2	0.90	0.86
	Core	Core
WHIM18	0.67	0.70
WHIM20	0.73	0.74
WHIM14	0.88	0.84
WHIM2	0.60	0.65

C

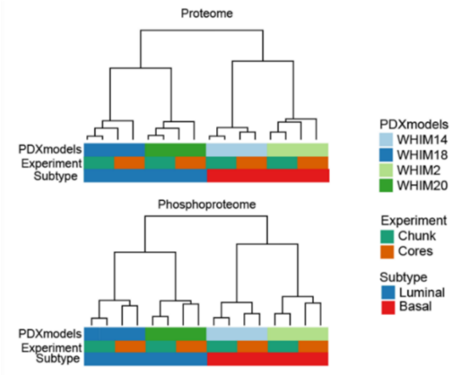

D

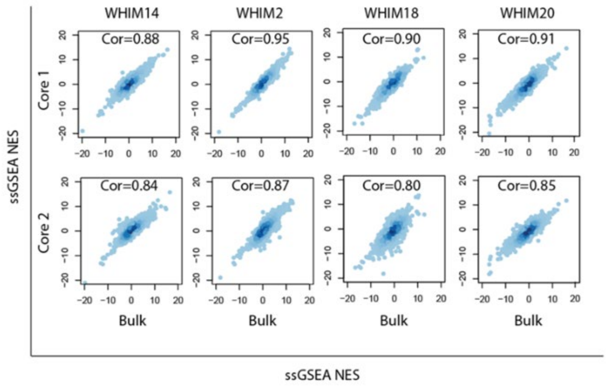

Supplementary Figure 2. Comparison of proteomics and phosphoproteomics dataset from tumor bulk and core samples. **A.** The table shows the number of proteins and phosphosites quantified in the bulk tissue (upper panel) and non-adjacent (lower panel) cores from 4 WHIM PDX models **B.** The table lists the Pearson correlation between replicate bulk and non-adjacent cores for each of the PDX models **C**. Unsupervised hierarchical clustering (1-Pearson) of normalized TMT protein and phosphosite ratios. **D**. ssGSEA was performed on normalized TMT protein ratios obtained from cores and bulk. Scatter plot shows ssGSEA normalized enrichment scores (NES) between cores and bulk tissue for individual PDX models.

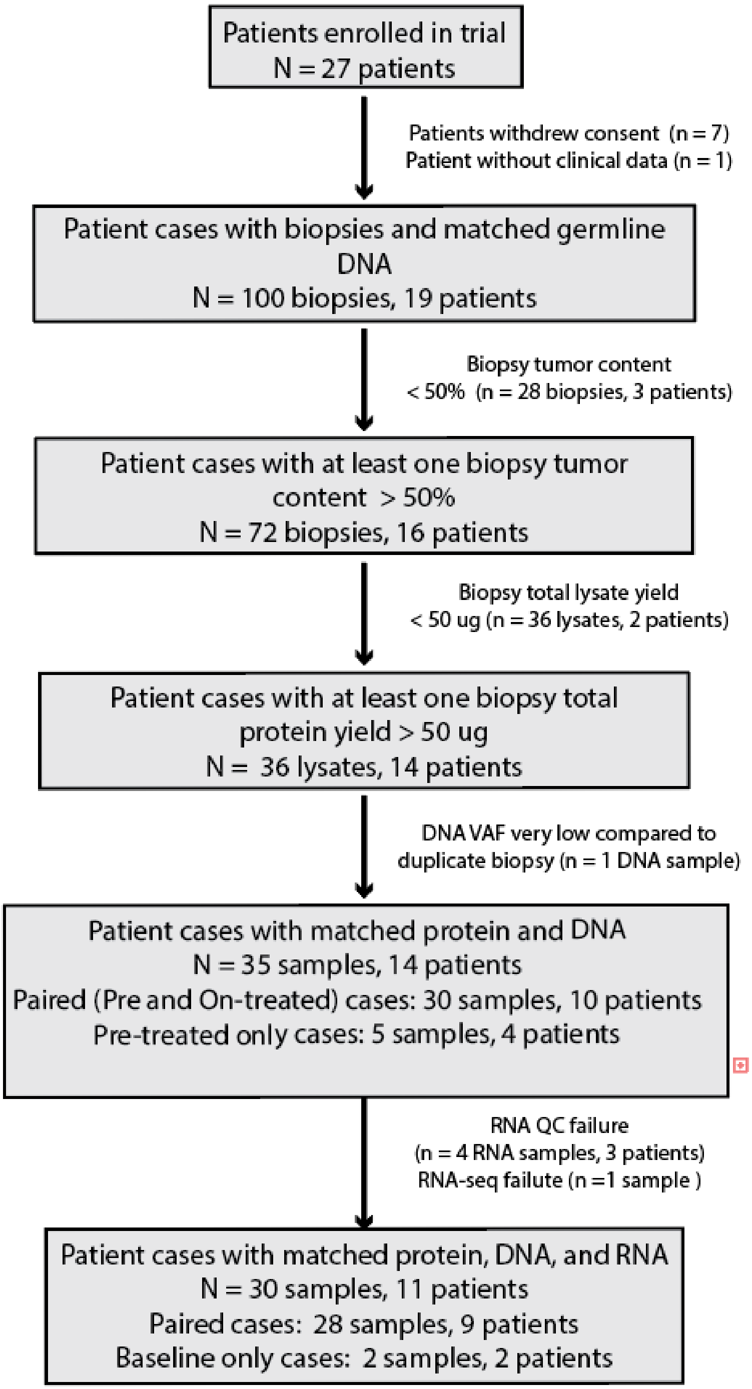

Supplementary Figure 3. Reporting Recommendations for Tumor Marker Prognostic Studies (REMARK) diagram. The flowchart shows the number of patients enrolled in the trial and reasons for their exclusion from the proteogenomic analysis when applicable.

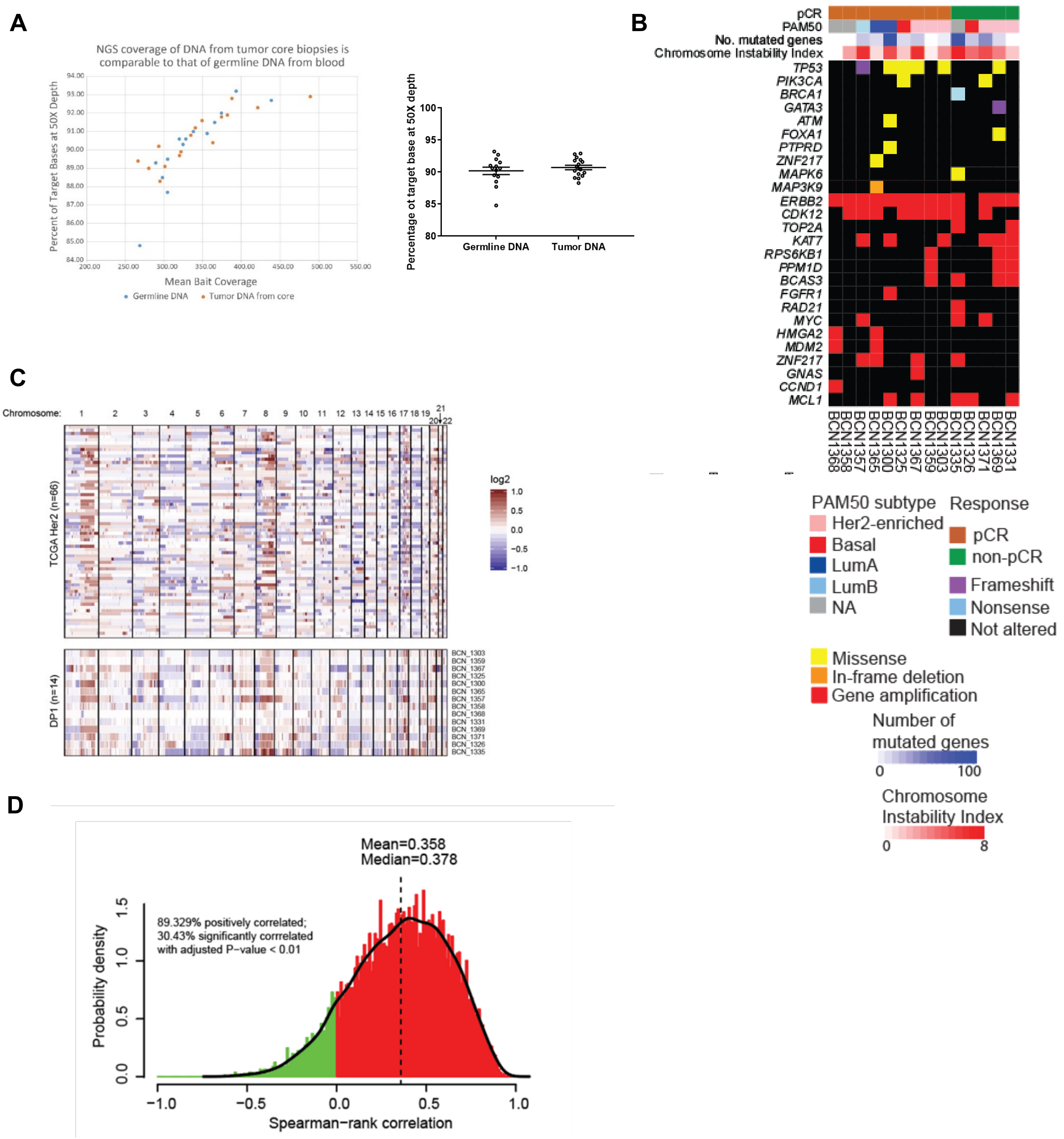

Supplementary Figure 4. Proteogenomics features of the clinical cores. **A.** The left panel shows a scatter plot of the percentage of target bases at 50X sequencing depth and of mean bait coverage for whole-exome sequencing and the right panel shows comparable mean distribution of the percentage of target bases at 50X sequencing depth for DNA isolated from blood versus that obtained from tumor using BioTExt The error bars represent standard error of mean. **B.** Heatmap summarizing genomic alterations of breast cancer associated genes in tumors from 14 patients. **C**. The copy number landscape of ERBB2+ samples from TCGA (top) resembles the landscape from this study (bottom). Plots show log2 ratios of chromosome segment copy number in tumor DNA relative to normal DNA for each patient (rows) from each cohort. **D**. Distribution of gene-wise Spearman correlations between RNA and Protein as observed using the BioTExt pipeline. Red and green indicate all positively and negatively correlated genes respectively.

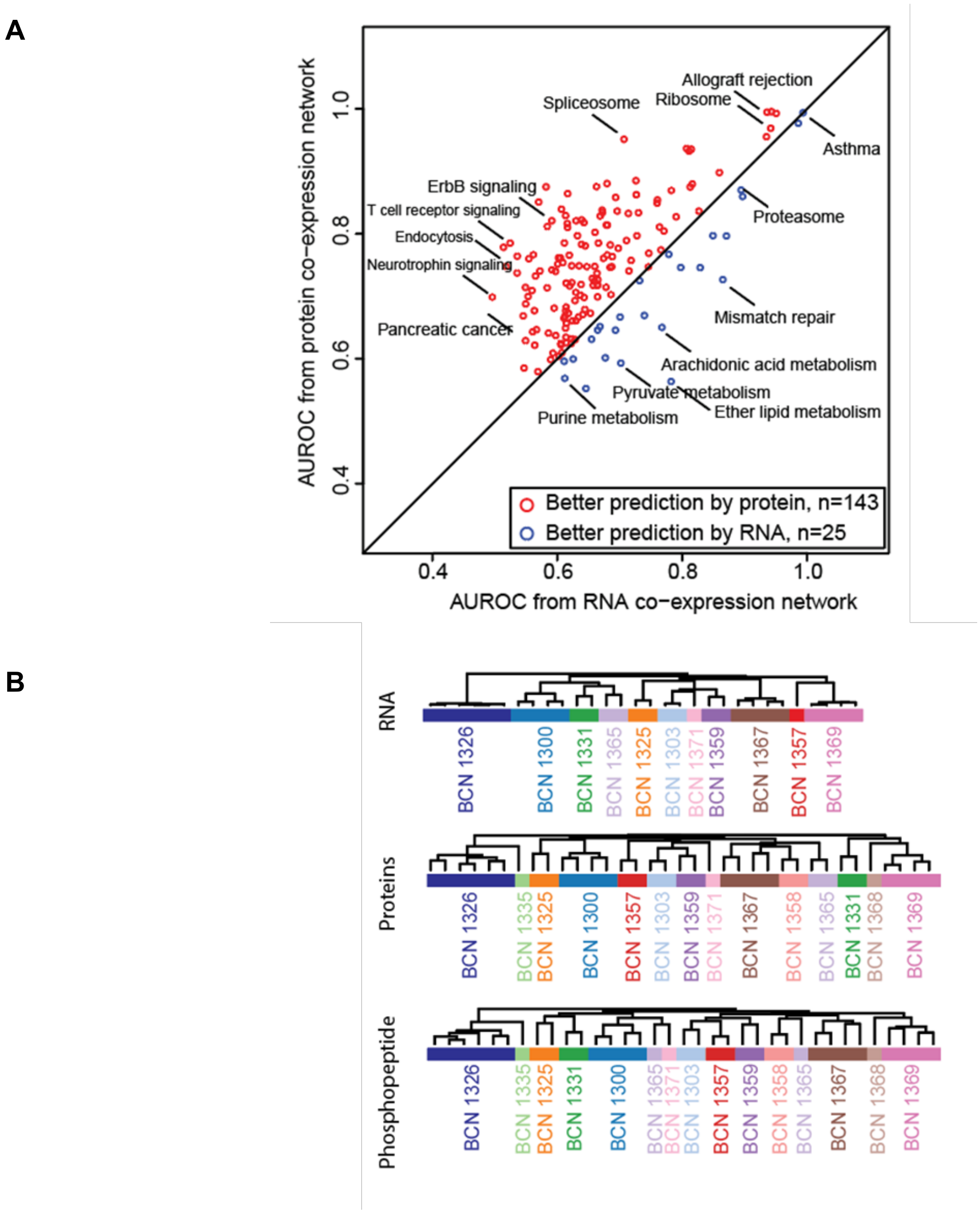

Supplementary Figure 5. Functional prediction from co-expression networks derived from and unsupervised hierarchical clustering of samples from clinical core proteogenomics data. **A**. Co-expression networks derived from microscaled proteomics data predict function more consistently than co-expression networks derived from the RNA data. Red and blue circles indicate functional categories (KEGG pathways) predicted by co-expression networks derived from protein and mRNA expression data, respectively. **B**. Core needle biopsies from the same patients cluster together based on the top 500 most variable features in each dataset.

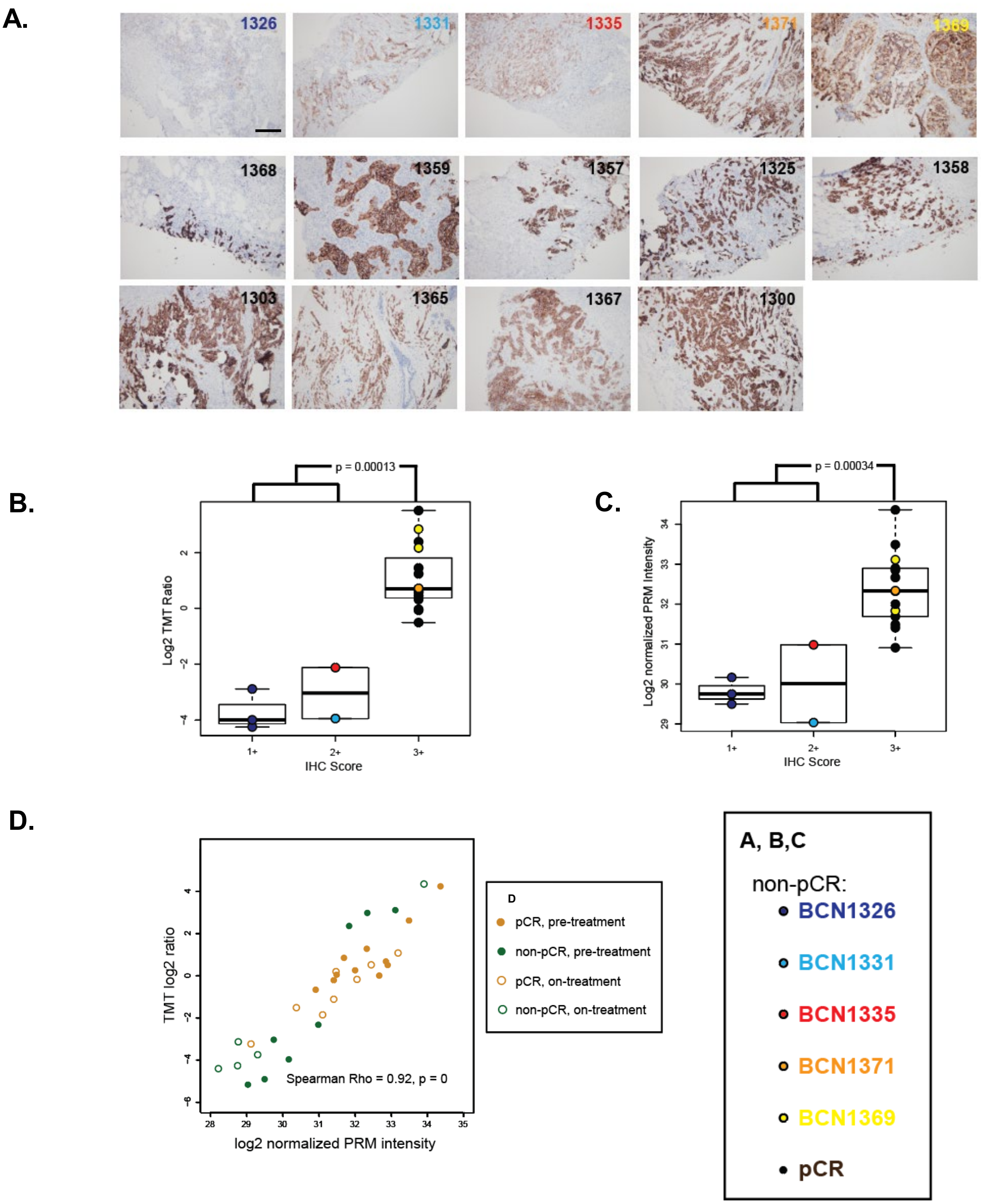

Supplementary Figure 6. Validation of ERBB2 levels. A. ERBB2 (HER2) immunohistochemistry (IHC) on sections from all 14 patients. **A**. Photomicrographs showing ERBB2 IHC staining profiles of all pCR cases at 200X. Scale bar represents 200 um for all. **B**. Box plot showing ERBB2 IHC scores and ERBB2 protein levels. *P*-value is from Wilcoxon two-sample rank sum test comparing pre-treatment log2 TMT ratios from IHC 1+ and 2+ samples (*n*=5) to those from IHC 3+ samples (*n*=15). **C**. Box plot showing ERBB2 IHC scores and ERBB2 protein levels as measured by parallel reaction monitoring (PRM). *P*-value is from Wilcoxon two-sample rank sum test comparing pre-treatment log2 PRM intensities from IHC 1+ and 2+ samples (*n*=5) to those from IHC 3+ samples (*n*=14). **D**. Scatter plot showing correlation between ERBB2 protein abundance measured using TMT and PRM based protein quantification for all cores (statistics shown for Spearman correlation, *n*=32). Boxplots are centered on the median and show first and third quartiles for each group.

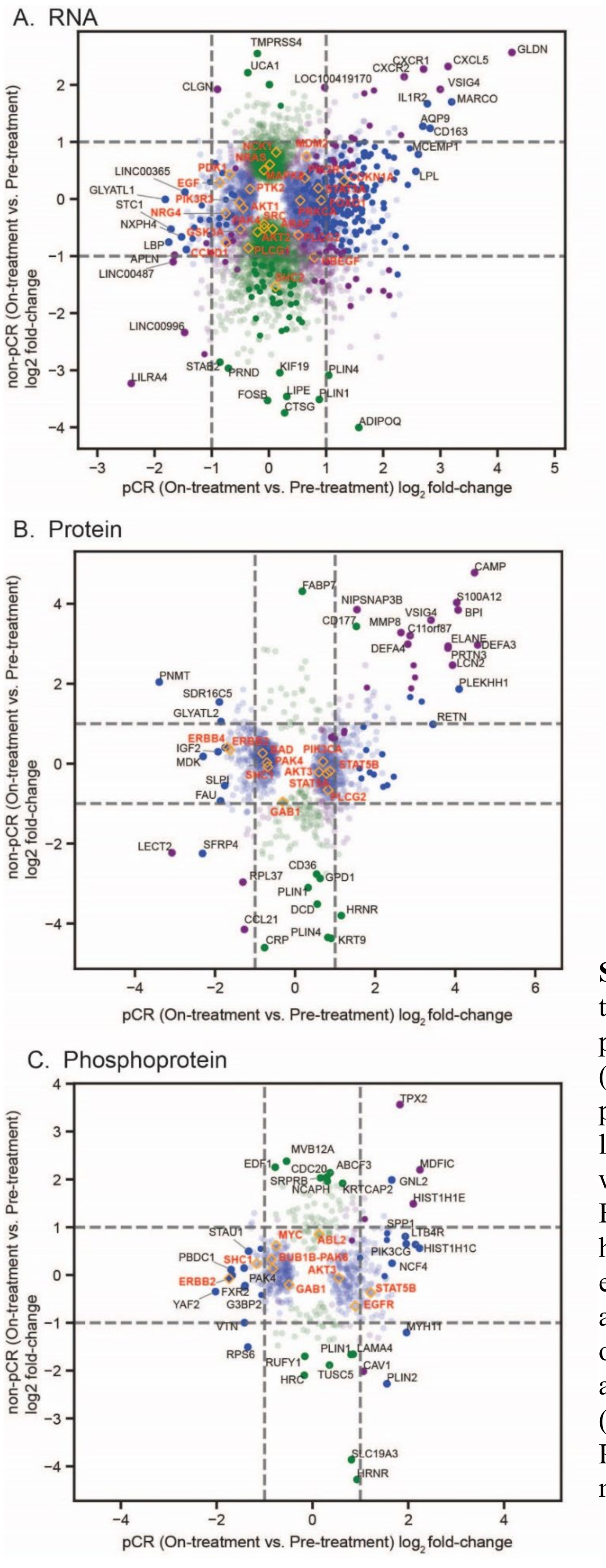

Supplementary Figure 7. Scatter plots of response to treatment (on-treatment vs pre-treatment) in non-pCR (y-axis) vs. pCR patients for RNA (**A**), proteins (**B**), and phosphoproteins (**C**) (mean of phosphosites). Shown are log2 ratios from *limma* linear modeling of differential expression for genes with $p<0.05$ in each set of patients. Genes from the ERBB signaling KEGG pathway (hsa04012) are highlighted in orange. The level of transparency of each point reflects it's significance after BH-adjustment (adjusted $p<0.05$ points are completely opaque, and more transparent points have higher adjusted p-values). $n=2$ for non-pCR samples (BCN1369 excluded because patient didn't receive Pertuzumab) and $n=7$ for pCR samples ($n=6$ for pCR mRNA).

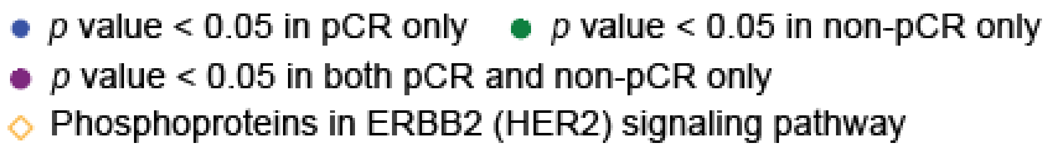

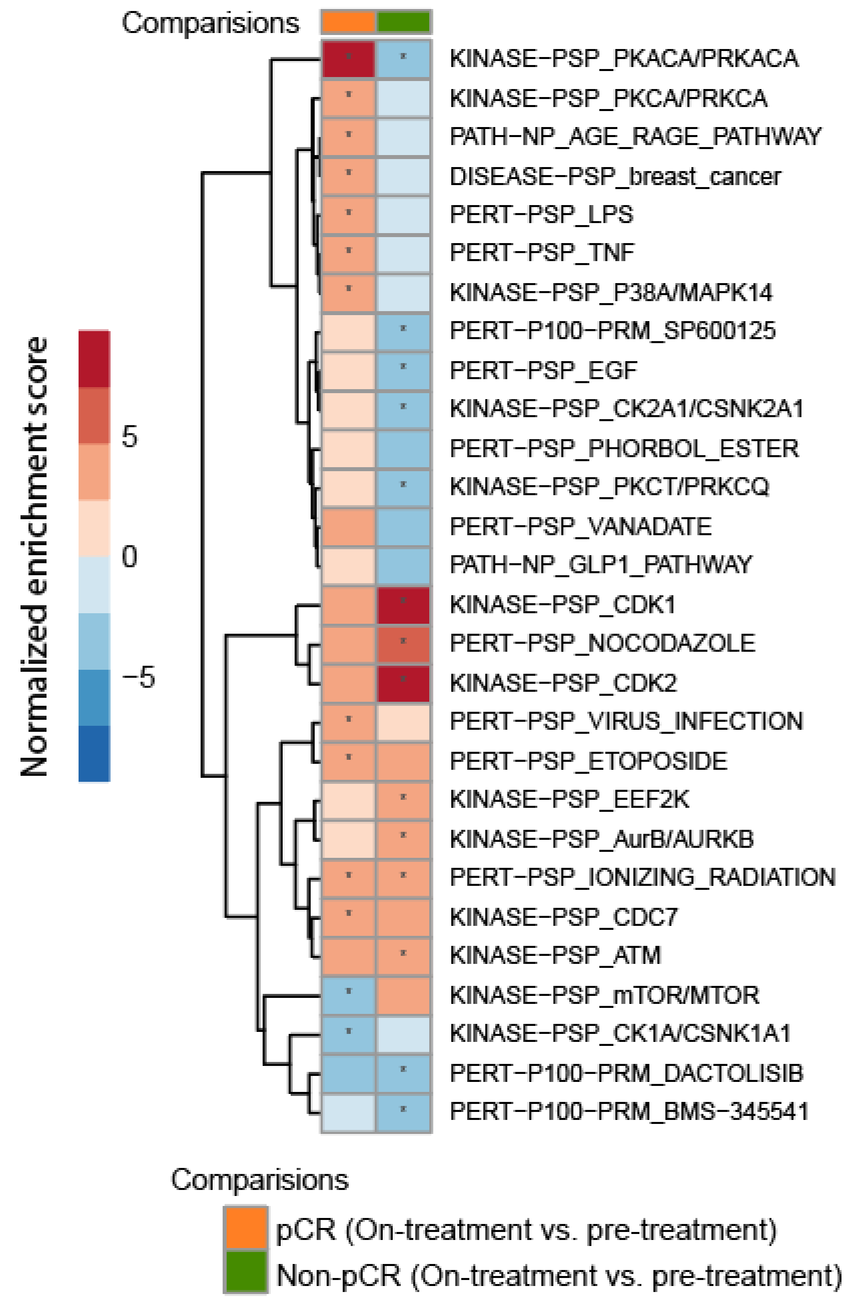

Supplementary Figure 8. PTM-SEA analysis on pre and on-treatment phosphoproteomics dataset. PTM-SEA was applied to the signed Log10 p-values from *limma* differential expression analysis of on- vs. pre-treatment phosphosite levels from pCR cases (orange) and non-pCR (green) The heatmap shows the Normalized Enrichment Scores (NES) for these kinase signatures, and asterisks indicate significant FDR (<0.05).

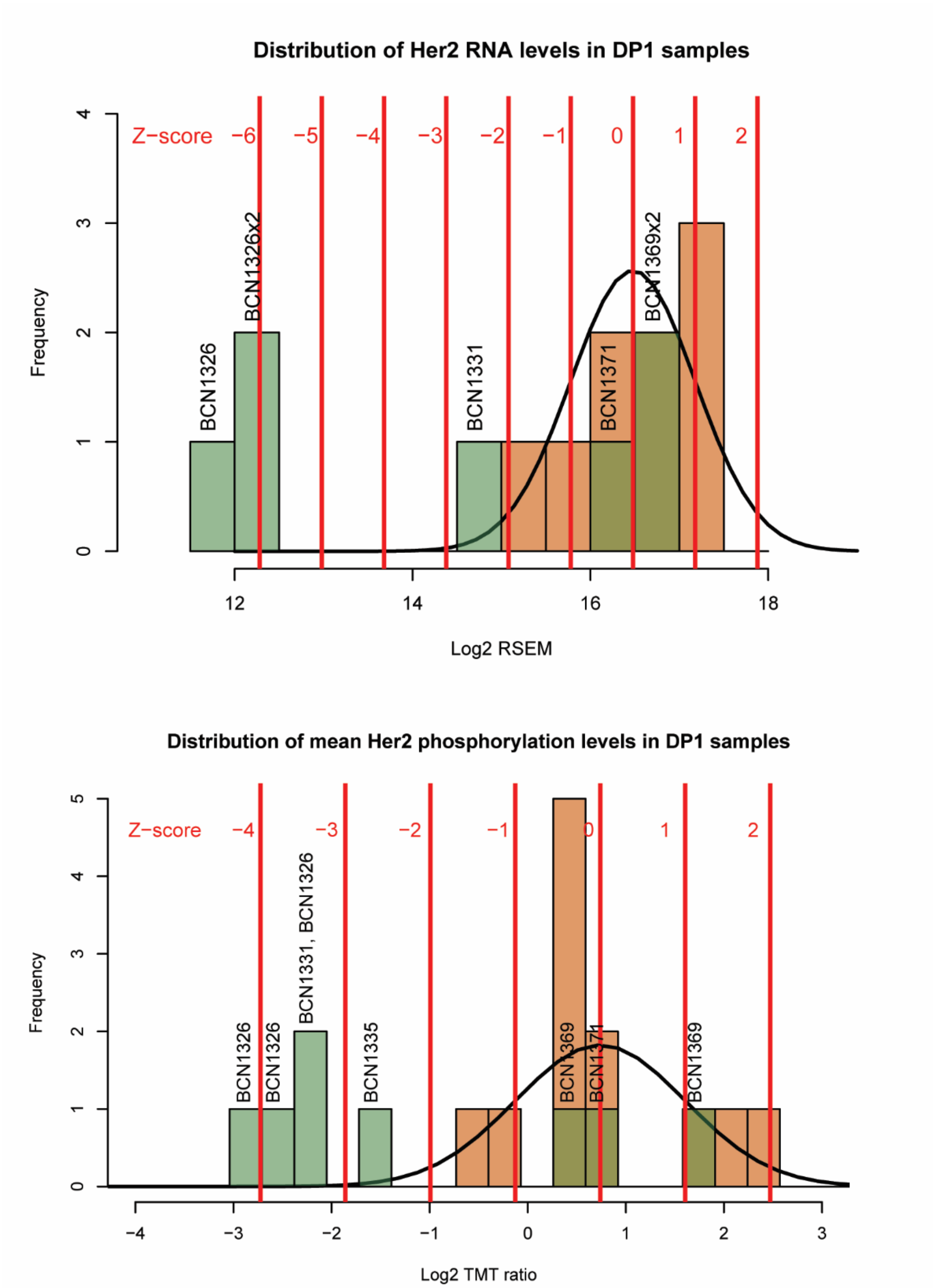

Supplementary Figure 9. Outlier analysis was performed to identify differentially regulated mRNA, proteins or phosphoproteins in each pre-treatment sample from non-pCR cases relative to the set of pre-treatment samples from all pCR cases. Shown are the ERBB2 (HER2) RNA (**A**) and phosphoprotein (**B**) distributions across all patients; brown and green bars indicate the frequencies for each protein level bin in non-pCR and pCR, respectively. The line shows the normal distribution of pCR samples from which the *Z*-score for each non-pCR sample was derived. *Z*-score thresholds are indicated by red lines.

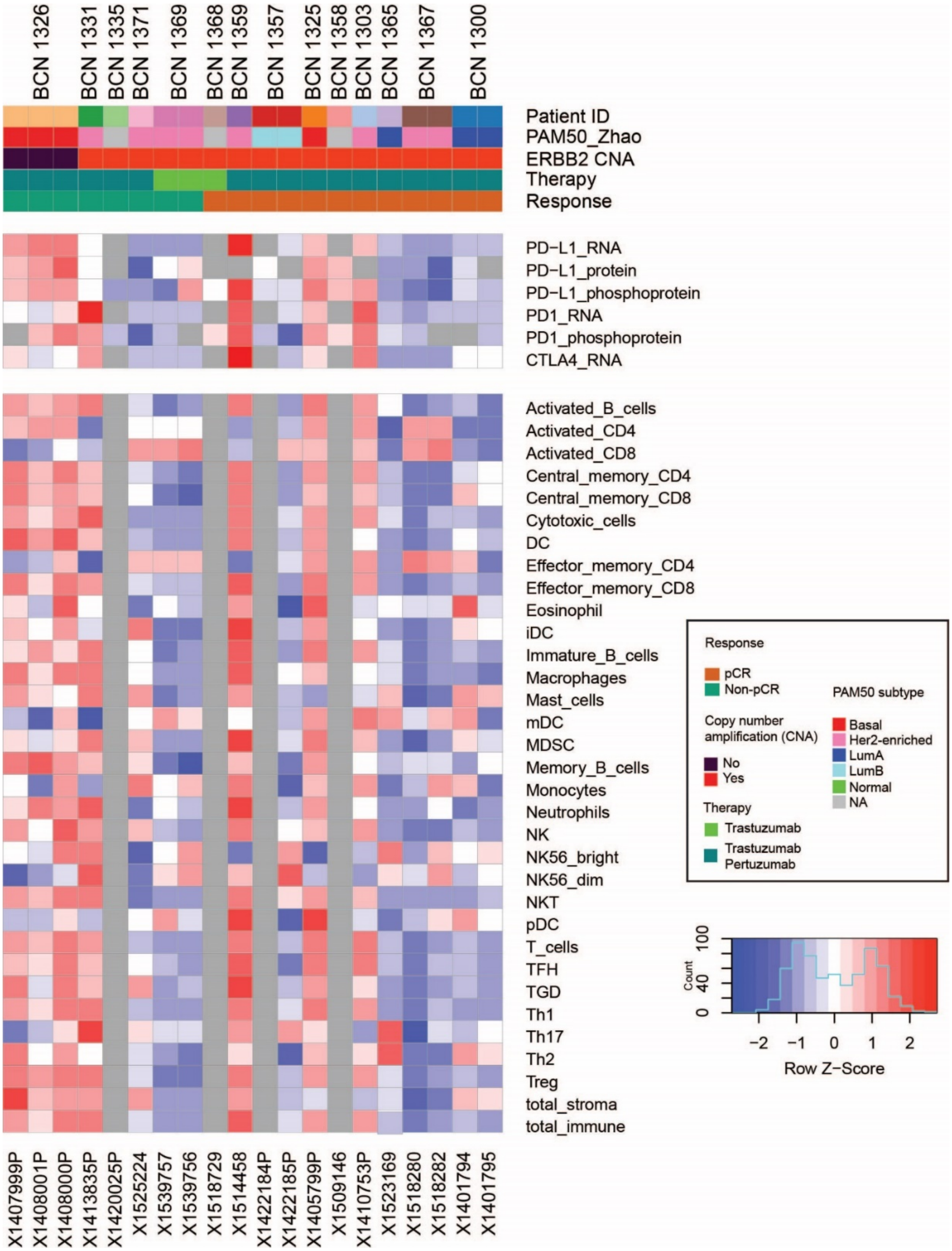

Supplementary Figure 10. Expression of key immune checkpoint regulators in and immunoprofiling of pre-treated samples. Upper panel shows *Z*-scores of RNA, protein, and phosphoprotein expression (where available) of key immune checkpoint inhibitors in each baseline sample from pCR (samples on right) and non-pCR (samples on left) patients. Bottom panel shows *Z*-scores of immune cell profiles inferred from RNA-seq data using Cibersort.

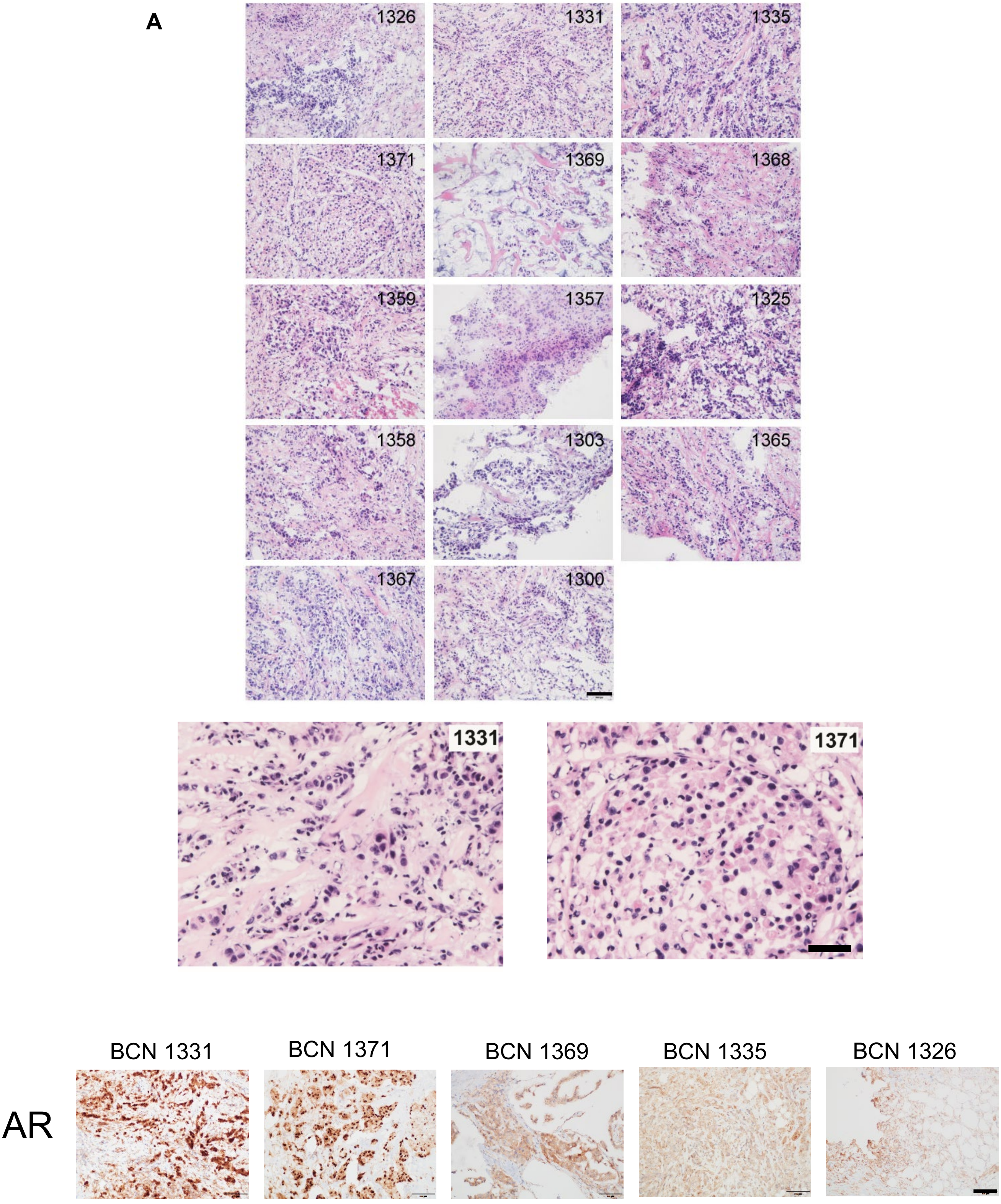

Supplementary Figure 11. Hematoxylin and eosin (HE) staining of patient samples. HE staining of tissue sections from all 14 patients and scale bar represents 100 um for all panels as shown in one representative.. The middle panel shows magnified HE stains (400X) of sections from AR+ patients 1331 and 1371 and scale bar represents 50 um. Patient 1371 shows distinct apocrine features as indicated by plump pink cytoplasm. The lower panel shows immunohistochemical staining profiles of non-pCR cases for AR at x200 and scale bar represents 100 um

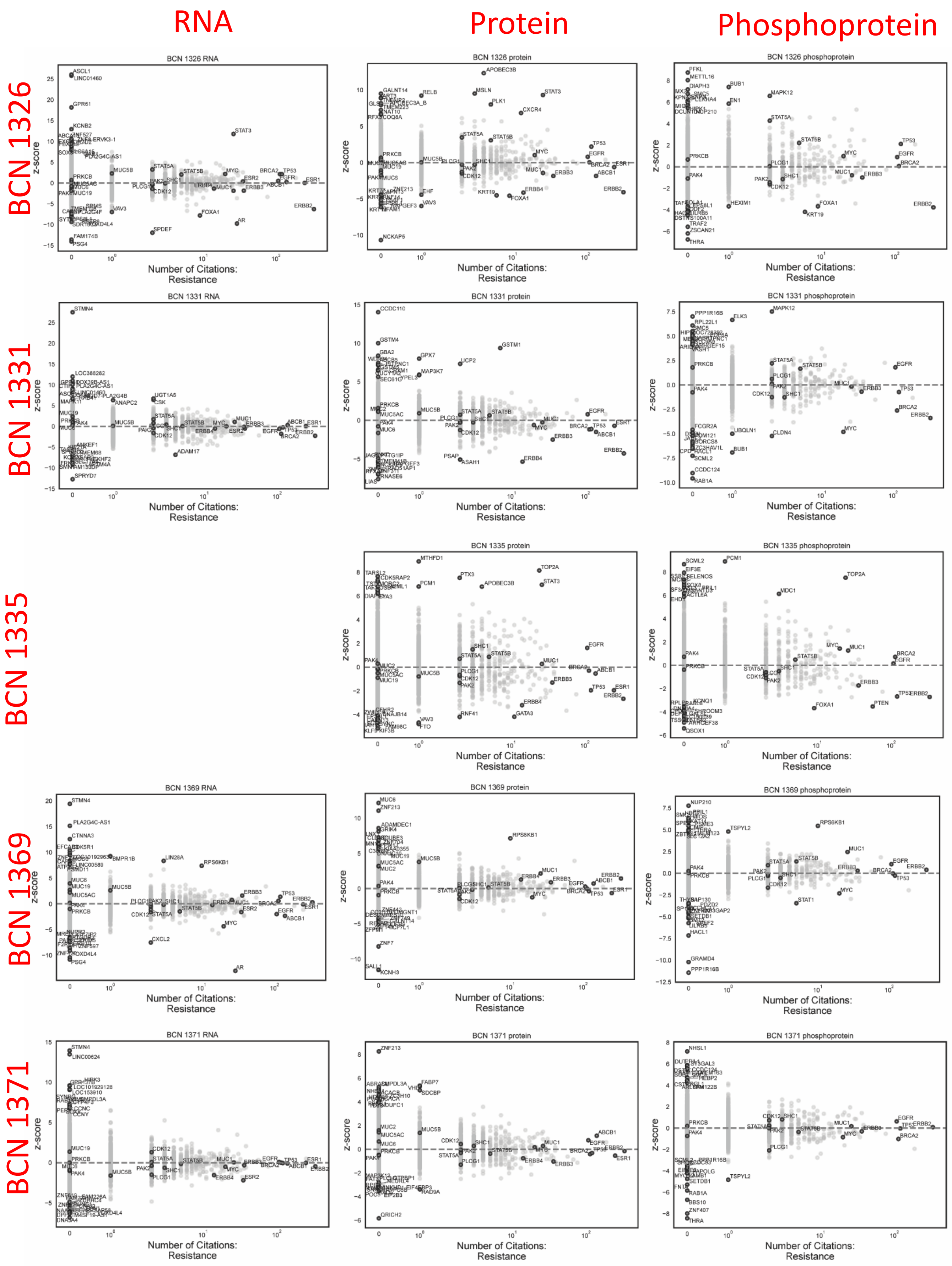

Supplementary Figure 12. Association of outliers with publications containing the keywords "breast cancer" and "resistance" or "recur". *Z*-score for each gene from outlier analysis is plotted on the y-axis, while the x-axis indicates the number of publications associated with that gene and with breast cancer resistance terms. A separate plot is included for outliers for each non-pCR sample from each omics dataset.

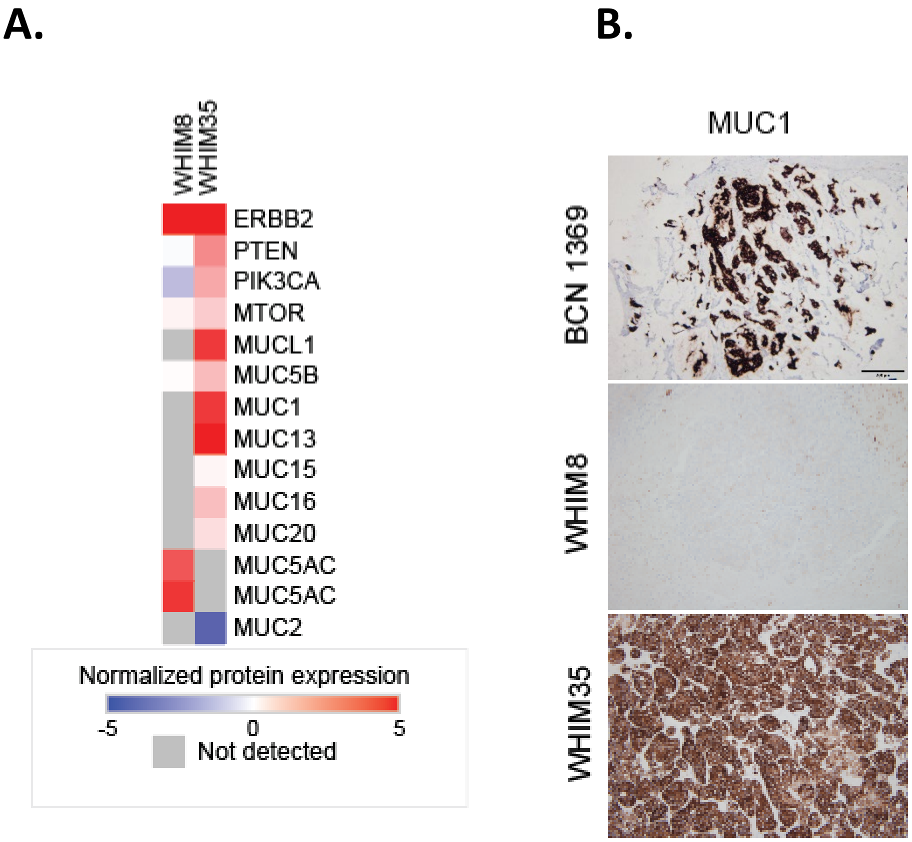

Supplementary Figure 13. ERBB2 and MUCIN expression in WHIM35, WHIM8 and patient BCN1326. **A**. Heatmap showing ERBB2 pathway and Mucin protein expression in two HER2-enriched PDX (WHIM) models with ERBB2 protein expression. **B**. MUC1 immunohistochemistry (IHC) of WHIM8, WHIM35 and BCN1369 and scale bar represents 200 um for all of B
