# Supplementary material for: Microscaled proteogenomic methods for precision oncology
Source: Nat Commun. 2020 Jan 27;11:532. doi: 10.1038/s41467-020-14381-2 (PMC6985126; doi:10.1038/s41467-020-14381-2)
Supplement: Supplementary file 2 — Reporting Summary [file 41467_2020_14381_MOESM2_ESM.pdf]

nature research

Corresponding author(s): Matthew J. Ellis, Steven Carr

Last updated by author(s): Nov 5, 2019

Reporting Summary

Nature Research wishes to improve the reproducibility of the work that we publish. This form provides structure for consistency and transparency in reporting. For further information on Nature Research policies, see Authors & Referees and the Editorial Policy Checklist.

Statistics

For all statistical analyses, confirm that the following items are present in the figure legend, table legend, main text, or Methods section.

n/a	Confirmed	
☐	☒	The exact sample size (*n*) for each experimental group/condition, given as a discrete number and unit of measurement
☐	☒	A statement on whether measurements were taken from distinct samples or whether the same sample was measured repeatedly
☐	☒	The statistical test(s) used AND whether they are one- or two-sided<br>*Only common tests should be described solely by name; describe more complex techniques in the Methods section.*
☒	☐	A description of all covariates tested
☐	☒	A description of any assumptions or corrections, such as tests of normality and adjustment for multiple comparisons
☐	☒	A full description of the statistical parameters including central tendency (e.g. means) or other basic estimates (e.g. regression coefficient) AND variation (e.g. standard deviation) or associated estimates of uncertainty (e.g. confidence intervals)
☐	☒	For null hypothesis testing, the test statistic (e.g. *F*, *t*, *r*) with confidence intervals, effect sizes, degrees of freedom and *P* value noted<br>*Give P values as exact values whenever suitable.*
☒	☐	For Bayesian analysis, information on the choice of priors and Markov chain Monte Carlo settings
☒	☐	For hierarchical and complex designs, identification of the appropriate level for tests and full reporting of outcomes
☐	☒	Estimates of effect sizes (e.g. Cohen's *d*, Pearson's *r*), indicating how they were calculated

Our web collection on statistics for biologists contains articles on many of the points above.

Software and code

Policy information about availability of computer code

Data collection	Orbitrap Fusion Lumos 3.0 Tune
Data analysis	R version 3.5.0, Microsoft Excel 2016, ssGSEA 2.0, VarScan, ANNOVAR, GISTIC, ESTIMATE, Cibersort, xCell

For manuscripts utilizing custom algorithms or software that are central to the research but not yet described in published literature, software must be made available to editors/reviewers. We strongly encourage code deposition in a community repository (e.g. GitHub). See the Nature Research guidelines for submitting code & software for further information.

Data

Policy information about availability of data

All manuscripts must include a data availability statement. This statement should provide the following information, where applicable:
- Accession codes, unique identifiers, or web links for publicly available datasets
- A list of figures that have associated raw data
- A description of any restrictions on data availability

Genomics data associated with this study will be available via dbGAP with dbGAP study id is phs001907.v1 and proteomics data is available via the CPTAC portal (https://cptac-data-portal.georgetown.edu/cptac/s/S051)

Field-specific reporting

Please select the one below that is the best fit for your research. If you are not sure, read the appropriate sections before making your selection.

☒ Life sciences ☐ Behavioural & social sciences ☐ Ecological, evolutionary & environmental sciences

October 2018

Life sciences study design

All studies must disclose on these points even when the disclosure is negative.

Sample size	No statistical testing was performed to determine sample due to the nature of the study
Data exclusions	See Supplemental Figure 3 REMARK
Replication	As described in the paper, several genomics and proteomics findings were validated using immuno-histochemistry and all replication attempts have been successful.
Randomization	no randomization
Blinding	This was not a blinded study.

Reporting for specific materials, systems and methods

We require information from authors about some types of materials, experimental systems and methods used in many studies. Here, indicate whether each material, system or method listed is relevant to your study. If you are not sure if a list item applies to your research, read the appropriate section before selecting a response.

Materials & experimental systems

n/a	Involved in the study	
☐	☒	Antibodies
☒	☐	Eukaryotic cell lines
☒	☐	Palaeontology
☐	☒	Animals and other organisms
☐	☒	Human research participants
☐	☒	Clinical data

Methods

n/a	Involved in the study	
☒	☐	ChIP-seq
☒	☐	Flow cytometry
☒	☐	MRI-based neuroimaging

Antibodies

Antibodies used	See methods section: Immunohistochemistry (IHC)
Validation	Muc1 (sc-7313); Santa Cruz: https://www.scbt.com/scbt/product/mucin-1-antibody-vu4h5<br>AR 441 (sc-7305); Santa Cruz: https://www.scbt.com/scbt/product/ar-antibody-441<br>Her2, cat#RM-9103-S-A: https://www.thermofisher.com/order/catalog/product/RM-9103-S-A?SID=srch-hj-RM-9103-S-A<br>CD3, cat#A045201-2: https://www.agilent.com/store/productDetail.jsp?catalogId=A045201-2

Animals and other organisms

Policy information about studies involving animals; ARRIVE guidelines recommended for reporting animal research

Laboratory animals	SCID/bg mice (Envigo)
Wild animals	NA
Field-collected samples	NA
Ethics oversight	For PDX studies, all animal procedures were approved by the Institutional Animal Care and Use Committee at Baylor College of Medicine (Houston, TX, USA) (protocol# AN-6934)

Note that full information on the approval of the study protocol must also be provided in the manuscript.

Human research participants

Policy information about studies involving human research participants

Population characteristics	Provided as supplementary table 1B.
Recruitment	Patients were recruited in breast oncology clinics. The only bias could be the willingness of a patient to undergo repeat biopsy.
Ethics oversight	All subjects provided written consent to research according to a protocol approved by human subjects research boards at the

Ethics oversight	institutions where the patients were accrued. This study was also approved by an institutional review board committee at Broad Institute, Baylor School of Medicine and University of Washington at St. Louis, and the NSABP Foundation. Inc. All patients provided consent for proteogenomics analyses.

Note that full information on the approval of the study protocol must also be provided in the manuscript.

Clinical data

Policy information about clinical studies

All manuscripts should comply with the ICMJE guidelines for publication of clinical research and a completed CONSORT checklist must be included with all submissions.

Clinical trial registration	NCT01850628
Study protocol	Available from investigators listed in https://clinicaltrials.gov/ct2/show/NCT01850628
Data collection	Samples were accrued from oncology clinics at Washington University in St Louis and Baylor College of Medicine, Houston. The protocol accrued between 2014 and 2017
Outcomes	The primary clinical outcome is determination of the pathological complete response rate following neoadjuvant therapy (pCR or nonpCR).
