# Supplementary material for: Microscaled proteogenomic methods for precision oncology
Source: Nat Commun. 2020 Jan 27;11:532. doi: 10.1038/s41467-020-14381-2 (PMC6985126; doi:10.1038/s41467-020-14381-2)
Supplement: Supplementary file 3 — Description of Additional Supplementary Files [file 41467_2020_14381_MOESM3_ESM.docx]

**Description of Additional Supplementary Files**

File name: Supplementary Data 1
Description: Experimental layout and metadata associated with TMT10/11 proteomics experiments. **A**. Experimental layout used for the TMT10-proteomics analysis of the PDX bulk and core tumors. **B**. Experimental layout used for the TMT11-proteomics analysis of 35 clinical cores derived from a total of 14 patient.

File name: Supplementary Data 2
Description: Proteomics datasets obtained from 4 WHIM PDX models and ssGSEA and PTM-SEA analysis on proteomics and phosphoproteomics datasets respectively. **A.** List of proteins identified from tumor bulk and cores derived from 4 PDX models (WHIM18, WHIM20, WHIM2 and WHIM4). Also included are the log2 transformed and median-MAD normalized TMT ratios for human proteins. **B**. List of phosphosites identified from tumor bulk and cores derived from 4 PDX models (WHIM18, WHIM20, WHIM2 and WHIM4). Also included are the log2 transformed and median-MAD normalized TMT ratios for individual human phosphosites. **C.** List of MSigDB “Hallmark” signatures from ssGSEA analysis performed on signed log p-values from *limma* analysis of differential protein expression between basal and luminal bulk or core tumor tissue. **D.** List of PTM-SEA signatures from PT-SEA analysis performed on signed log p-values from *limma* analysis of differential phosphosite expression between basal and luminal bulk or core tumor tissue.

File name: Supplementary Data 3
Description: Genomics datasets obtained from 14 patients characterized in this study. **A**. Binary table of somatic mutations affecting protein sequence in 14 pre-treated core needle biopsies. Mutations are listed in format provided by ANNOVAR (<HGNC_gene>:<ref_seq_ID>:<nucleotide_changes>:<amino_acid_changes>), and each sample was assigned a 1 or 0 to indicate whether the mutation was present in the tumor. **B**. Binary table of somatically mutated genes from 14 pre-treated core needle biopsies. Mutated genes are indicated as 1 and non-mutated genes are indicated as 0. **C**. Table of copy number aberration (CNA) data from GISTIC2 showing actual changes in copy number (log2 ratios) for each gene in 14 pre-treated core needle biopsies. **D**. Upper quantile normalized RSEM expected counts from RNA-seq data for 30 core needle biopsies, corresponding to 11 patients.

File name: Supplementary Data 4
Description: Proteomics dataset obtained from 14 patients characterized in this study. **A**. List of proteins identified from 35 core needle biopsies obtained from a total of 14 patients. Also included are the log2 transformed and median-MAD normalized TMT ratios. **B**. List of phosphosites identified from 35 core needle biopsies obtained from a total of 14 patients. Also included are the log2 transformed and median-MAD normalized TMT ratios. **C**. List of phosphoproteins identified from 35 core needle biopsies obtained from a total of 14 patients. TMT ratio for a phosphoprotein is the average of all log2 transformed and median-MAD normalized phosphosite ratios for a given gene **(from 4B)**

File name: Supplementary Data 5
Description: Fold-changes and p-values derived from *limma* analysis of response to treatment (on-treatment vs. pre-treatment) for pCR, non-pCR, and pCR vs. non-pCR samples (difference between response in pCR and non-pCR samples). Only samples from patients with both on- and pre-treatment cores were included in this analysis, and BCN1369 was excluded because she didn’t receive Pertuzumab. **A**. Fold-changes and p-values derived from gene-centric RNA-seq, proteome and phosphoproteome analyses. For gene level phosphoproteome analyses, data was aggregated by mean of all sites for each gene. **B**. Fold-changes and p-values derived from differential phosphosite analyses. Phosphosites were aggregated to the level of individual sites by using the mean for all phosphopeptides containing high confidence localization of each site from Supplementary Data 4B. Also included for A. and B. are the BH corrected p-values and logP-values signed by the direction of the change in expression.

File name: Supplementary Data 6
Description: List of kinase signatures from PTM-SEA analysis performed on signed log p-values for phosphosite level data from *limma* analysis of differential expression for response to treatment (from **5B**). Also included are the corresponding FDR and normalized enrichment scores (NES).

File name: Supplementary Data 7
Description: Outlier *Z*-scores and single-sample GSEA (ssGSEA) normalized enrichment scores. **A**. RNA, Protein and Phosphoprotein (mean of all phosphosites for each gene) outlier *Z* scores for genes in all of the pre-treated non-pCR core needle biopsies relative the distribution for the corresponding data in the set of pre-treated pCR biopsies. **B**. List of gene-sets from ssGSEA analysis performed on outlier *Z*-scores (from **5A**). Also included are the corresponding FDR filtered (FDR<0.25) normalized enrichment scores (NES).

File name: Supplementary Data 8
Description: Listed are the output from Pubmed crawler algorithm.
